# Automation, workers’ skills and job satisfaction

**DOI:** 10.1371/journal.pone.0242929

**Published:** 2020-11-30

**Authors:** Henrik Schwabe, Fulvio Castellacci

**Affiliations:** TIK Centre, University of Oslo, Oslo, Norway; University of Jyvaskyla, FINLAND

## Abstract

When industrial robots are adopted by firms in a local labor market, some workers are displaced and become unemployed. Other workers that are not directly affected by automation may however fear that these new technologies might replace their working tasks in the future. This fear of a possible future replacement is important because it negatively affects workers’ job satisfaction at present. This paper studies the extent to which automation affects workers’ job satisfaction, and whether this effect differs for high- versus low-skilled workers. The empirical analysis uses microdata for several thousand workers in Norway from the *Working Life Barometer* survey for the period 2016–2019, combined with information on the introduction of industrial robots in Norway from the International Federation of Robotics. Our identification strategy exploits variation in the pace of introduction of industrial robots in Norwegian regions and industries since 2007 to instrument workers’ fear of replacement. The results indicate that automation in industrial firms in recent years have induced 40% of the workers that are currently in employment to fear that their work might be replaced by a smart machine in the future. Such fear of future replacement does negatively affect workers’ job satisfaction at present. This negative effect is driven by low-skilled workers, which are those carrying out routine-based tasks, and who are therefore more exposed to the risks of automation.

## 1. Introduction

Industrial robotics and artificial intelligence (AI) have in the last few years increasingly been used in production activities. This has led to the automation of many tasks that were previously carried out by workers, and that can now be performed by smart machines. The fear that these technological advances may have dramatic consequences on the future of labor has fostered the recent development of new economics research studying the effects of automation on employment [1,2]. Recent models and empirical evidence on this topic show that automation can have negative effects on employment demand and wages, and particularly so for workers that perform routine-based tasks that can more easily be displaced [3,4]. On the other hand, however, these new technologies may also have positive effects by increasing productivity [5].

This recent research has so far focused on the effects of automation, industrial robots and artificial intelligence on labor demand and wages. However, while employment and wages are two central dimensions shaping individual workers’ well-being, it is also important to point out that other non-pecuniary aspects do contribute to shape workers’ well-being, and that automation may potentially have important impacts on these [6]. Specifically, if workers fear that their occupation might be replaced by a smart machine in the future, such prospect and uncertainty about future working conditions may arguably affect their job satisfaction at present [7,8].

Why should we care about the impacts of automation on workers’ job satisfaction? The reason is twofold. First, since individuals spend a substantial part of their life at work, job satisfaction experienced in working life does indeed represent an important component of individuals’ overall subjective well-being [9]. Second, workers that are not happy and experience dissatisfaction with their job have typically lower motivation and efforts [10], and higher turnover rates. Therefore, if a large number of workers in the economy fear to be replaced by smart machines in the future, this fear may lead to mental stress and anxiety at present, as well as hamper productivity and innovation in the economy.

In spite of the relevance of this topic, to the best of our knowledge only two papers have recently explored the relationship between automation and workers’ well-being. Abeliansky and Beulmann [11] focuses on workers’ mental health in Germany; and Schwabe [12] studies workers’ life satisfaction in a sample of European countries. Neither of these studies, though, investigates explicitly the impacts of automation on job satisfaction.

Further, these recent works do not study the role of workers’ skills, and how these may affect the relationship between automation and well-being. The literature on automation and employment clearly shows that the effects of the introduction of industrial robots largely differ for high-skilled and low-skilled workers. It is therefore paramount to investigate whether the effects of automation on job satisfaction can have different effects on workers’ well-being depending on their skill levels. In short, the question investigated in the present paper is the following: *Does automation affect workers’ job satisfaction–and how does this effect differ for high- versus low-skilled workers*?

To study this question, it is useful to distinguish two related dimensions. The first side of the link between automation and job satisfaction is that the introduction of industrial robots in local labor markets will affect workers’ expectations about their future jobs, i.e. it will lead some workers to fear that part of their working tasks might be replaced by a smart machine in the future. The second dimension is that these expectations about the future, and particularly the anticipated fear of replacement, will negatively affect workers’ subjective well-being at present.

Empirically, we operationalize this idea by making use of a two-stage econometric model, in which fear of replacement and job satisfaction are the dependent variables of the first and the second stage, respectively. The empirical analysis uses microdata for several thousand workers in Norway from the *Working Life Barometer* survey (*Arbeidslivsbarometer*) (four annual surveys for the period 2016–2019), combined with information on the introduction of industrial robots in Norway from the *International Federation of Robotics* (IFR) dataset. Our identification strategy exploits variation in the pace of introduction of industrial robots in Norwegian regions and industries between 2007 and *t* (i.e. the time at which each of the four surveys was carried out).

The results indicate that automation in industrial firms in recent years has induced workers that are currently in employment to fear that their work might be replaced by a smart machine in the future, and that this effect is stronger for low-skilled workers. Further, our findings show that fear of future replacement does negatively affect workers’ job satisfaction at present, and that such negative effect is in particular significant for low-skilled workers, which are those carrying out routine-based tasks, and who are therefore more exposed to the risks of automation.

On the whole, these results contribute to, and extend, the recent literature on automation and employment by shifting the focus to important nonpecuniary impacts that are reflected in workers’ expectations, fears and job satisfaction, and showing that workers’ skills is an important variable moderating the effects of automation on subjective well-being.

The paper is organized as followed. Section 2 reviews the literature on automation and employment. Section 3 points out the conceptual mechanisms that are relevant to explain the effects of automation on job satisfaction. Section 4 presents the data and indicators. Section 5 discusses the empirical methods. Section 6 presents the results. Section 7 concludes and discusses the main contributions and implications.

## 2. Literature

### Effects of automation on employment and wages

Automation, industrial robotics and artificial intelligence have in the last few years experienced substantial advances and found an increasing number of applications in production activities. Artificial intelligence and robotics have developed as two distinct scientific and technological fields for a long time, and only recently they have converged and cross-fertilized [13]. Frank et al. [2] presents relevant illustrations of this recent convergence, and it discusses challenges for research on the economic effects of AI and automation. This has spurred the recent development of a strand of scholarly research studying the effects of these new technologies on employment.

A starting point of this literature is the canonical model of skilled bias [14], according to which new skilled-bias technologies lead to polarization and increasing differences in employment opportunities and wages between skilled and unskilled workers. Sachs and Kotlikoff [15] present a simple framework in which smart machines substitute directly for young unskilled labor, whereas they are complementary to older skilled workers. Young unskilled workers experience lower wages, which in turn lead to lower saving and investments in human and physical capital–thus perpetuating and strengthening the gap between young unskilled and older skilled workers over time.

Such pessimistic prediction on the future of employment is however not shared by other works in this field. Taking a long-run historical perspective, Autor [16] and Mokyr et al. [1] argue that, as in other times in history, technological progress will lead to major structural changes in the quantity and content of work, but it will arguably not lead to a complete substitution of capital for labor. Houseman [17] provides empirical evidence that, although manufacturing employment in the US has declined since early 2000s, this is mainly explained by international trade and global competition effects, and there is weak support in the data for the argument that such decrease in employment is due to automation. More recently, McGuinness et al. [18] and Klenert et al. [19] present empirical studies that indicate that automation technologies and industrial robots have actually positive effects on employment. On the one hand, automation leads to a creative destruction process that may on the whole increase the overall demand for labor. On the other hand, it may contribute to reduce routine-based working tasks, which are typically monotonous and physically straining, thus improving the quality of work.

A more nuanced perspective that considers both negative and positive effects of automation on employment is presented by studies of the job polarization hypothesis. In short, the main idea of this research is that automation technologies complement highly skilled labor, explaining its expansion and wage growth in recent years in most advanced countries. On the other hand, middle-skilled workers are those more negatively affected by routine-biased technical change, because their tasks are relatively easier to automate. As for low-skilled workers, and particularly those employed in personal services occupations, these often perform manual and personal communication tasks that are not that easy to automate yet. Hence, the resulting pattern is that middle-skilled workers have in recent years shifted towards low-skilled employment occupations, which have consequently grown and experienced higher wages. All in all, this explains the observed increasing polarization in the job market, with the growth of employment and wages for high- and low-skilled workers, and a corresponding decline for middle-skilled occupations [3,4,16,20]. Beaudry et al. [21] argue however that the demand for high-skilled workers has declined after 2000 due to decreasing returns to investments in information and communication technologies (ICTs), and that high-skilled have then begun to compete for lower-skilled jobs. This study, though, is based on empirical evidence on ICT investments in general, and it does not focus specifically on the effects of AI and automation.

Acemoglu and Restrepo [22] present a theoretical framework that is useful to study both negative and positive effects of industrial robots on employment and wages. The model points out two contrasting effects of industrial automation: a *displacement* effect that negatively affects the demand for employment and the wages of workers that perform routine-based tasks; and a *productivity* effect that creates benefits for workers that perform non-routine tasks (in the automated sector as well as in other sectors and occupations of the economy). This study also presents empirical evidence that corroborates the model’s predictions on the effects of industrial robots on employment and wages in US manufacturing industries between 1990 and 2007. In line with evidence presented by other recent works [5,23,24], their results show that overall the displacement (negative) effect of the introduction of industrial robots has until now been stronger than the productivity (positive) effect.

### Effects of automation on job satisfaction

This recent strand of research has so far focused on the effects of automation, industrial robots and artificial intelligence on aggregate patterns of labor demand and wages for different countries and industries. However, research has not investigated yet the impacts that these new technologies may have on individual workers’ subjective well-being. Do workers fear that their occupation might be replaced by a smart machine in the future, and if so how does that prospect affect their current job satisfaction?.

Job satisfaction is the subjective well-being of workers (i.e. their own assessment of the well-being they experience at work). This is an obviously crucial dimension for economic analysis and policy. First, since individuals spend a substantial part of their life at work, job satisfaction experienced in working life represents an important component of individuals’ overall subjective well-being. Second, workers that are not happy and experience dissatisfaction with their job have typically lower motivation and efforts, and higher turnover rates. This, in turn, weakens productivity and innovation in the economy.

The literature on job satisfaction is wide-ranging, and it has extensively investigated a variety of factors that explain why some individuals report higher subjective well-being than others [7,8]. However, only a few studies have so far explicitly investigated the relationships between the widespread diffusion and application of digital technologies and job satisfaction [25]. Kaplan and Schulhofer-Wohl [6], using data from the American Time Use survey, discusses the nonpecuniary implications of changes in the occupational structure in the US in recent decades, i.e. the effects of these structural changes on different aspects of job satisfaction such as reported happiness, stress and meaning at work. The work indicates that the changing occupational structure has not only led to polarization in terms of skills and wages, but it has also determined substantial changes in workers’ feelings about the job they have and the tasks they perform.

Two recent papers explore the relationship between automation and workers’ well-being. Abeliansky and Beulmann [11] present an empirical study on the impact of automation on the mental health of workers (which is one important dimension reflecting stress and weak job satisfaction). The analysis uses individual-level data from the German Socioeconomic Panel for the period 2002–2014 linked to industry-level data on use of industrial robots in 21 manufacturing sectors in Germany. The results indicate that automation negatively affects workers’ mental health, and this effect is related to the fear of having lower wages and worse economic conditions in the future.

Schwabe [12] makes use of worker-level data from the Eurobarometer survey for European countries (period 2012–2017) to investigate the relationships between fear of replacement and workers’ subjective well-being (measured by life satisfaction, which is as well-known an evaluative dimension of individuals’ well-being). The results of this study find that fear of replacement affects life satisfaction, but the direction of this effect does largely depend on age. In line with models of skill-bias and job polarization (see section 2.1), younger workers regard replacement as a possible threat to their job opportunities in the future, whereas older workers look at it as a positive technological development that is not likely to affect them directly, and that will arguably enhance well-being and prosperity in the society.

These two studies provide an important starting point for the present work. None of them, though, investigates explicitly the role of workers’ skills, which is however a key dimension in the literature on the employment effects of automation briefly reviewed in section 2.1. In the job satisfaction literature too, education and skill levels represent one of the central factors affecting the job satisfaction of workers [26].

Two contrasting mechanisms link education and job satisfaction. On the one hand, a higher skill level increases the chances that an employee will have a higher wage level and a more interesting and rewarding job, which enhance job satisfaction. On the other hand, however, various empirical studies have found that–after controlling for income earnings–the correlation between education level and subjective well-being at work is negative [8,27,28]. This can be explained in the light of prospect theory [29]. When an individual invests more time in education and human capital formation, her expectations about the desired job will also be higher, and it will therefore be more likely that the worker will feel more critical and less satisfied with her actual working conditions if these high expectations are unmet. In particular, empirical research indicates that overqualified workers report significant lower levels of job satisfaction than others [26,30].

## 3. Question and propositions

The question investigated in the present paper is the following: *Does automation affect workers’ job satisfaction–and how does this effect differ for high- versus low-skilled workers*? The first part of the question refers to the main impact of automation on job satisfaction, which as noted above has not been analyzed in previous research yet. The second part of the question suggests that fear of replacement can have different effects on workers’ well-being depending on their skill levels, and it seeks to investigate these moderation effects.

Conceptually, the link between automation and job satisfaction can be analyzed in two steps. The first is that the introduction of industrial robots in local labor markets will arguably affect workers’ expectations about their future jobs, which means that some workers will fear that some of their tasks, or even their whole job, might be replaced by a smart machine in the future. The second step is that these expectations about the future, and particularly the anticipated fear of replacement, will affect workers’ job satisfaction at present.

Our empirical analysis will consider both of these conceptual steps in a two-stage empirical model, and investigate whether the related impacts are stronger for high-skilled or for low-skilled workers. We point out below here the main effects that we expect to find in the empirical analysis, and how these can be explained in the light of the literature reviewed in this section. As noted below, some of the effects of interest are stronger for high-skilled workers, whereas others are more relevant for low-skilled workers, so that the overall net moderation effect cannot be pointed out *ex-ante*, but it will have to be established based on the empirical evidence.

### I. Fear of replacement

The introduction of industrial robots in the local labor market increases the likelihood that some workers will be replaced by smart machines in the future. These technological changes and their applications in firms in local labor markets will therefore induce some workers that are currently employed to fear that they might be replaced in the future (or at least that some of their tasks might be).

#### Moderation effects

The introduction of industrial robots will arguably have different impacts for high- *versus* low-skilled workers. We envisage two contrasting effects.

*Fear of replacement is stronger for the low-skilled*. These workers are more exposed to the risks of displacement from automation because they typically carry out routine tasks that can more easily be automated (see literature in section 2.1).

*Fear of replacement is stronger for the high-skilled*. High-skilled workers are typically also more educated individuals who read more and follow media debates on robots, automation and their negative consequences for employment. Hence, high skilled workers are arguably more exposed to peer effects, which may translate in a greater fear about the future of employment. Contrary to this argument, we may however posit that workers of higher education typically have a better ability to understand and anticipate that these new technologies will also have positive effects for their future tasks and wages, as well as for the overall productivity of the economy–i.e. they are arguably be more forward-looking [31].

**Proposition 1:** The introduction of industrial robots in the local labor market will negatively affect low-skilled workers more than high-skilled workers if the former effect is stronger than the latter.

### II. Job satisfaction

The second aspect of our conceptual analysis refers to the impacts that fear of replacement will have for workers’ subjective well-being. The main expectation is that fear of replacement in the future will negatively affect job satisfaction at present. The main reason is that the prospect to become unemployed, or to be taken away some of the current working tasks, will negatively affect wage and financial conditions expected for the future, thus creating uncertainty about future job prospects and personal finance, and hence lower job satisfaction.

#### Moderation effects

Fear of replacement will arguably have different impacts on job satisfaction for high- versus low-skilled workers. We posit the following contrasting effects.

*The negative effects on job satisfaction will be stronger for the low-skilled*. If replaced, these workers will on average have fewer possibilities to find another occupation in the labor market. Acemoglu and Restrepo [22] and Blanas et al. [20] document in fact that displacement effects of industrial robots on employment and wages are stronger and more significant for low-education workers. On the other hand, as noted in section 2.1, extant research suggests that automation technologies can have more positive effects on high-skilled workers, increasing the demand for labor, wages and the complexity and interest of their tasks [18].

*The negative effects on job satisfaction will be stronger for the high-skilled*. According to prospect theory [29], individuals that invest more time in education and human capital formation will also have higher expectations about the working conditions that they desire and expect to have in the future, and be less satisfied with their job if this does not match the high expectations the individual has. Hence, highly educated workers, when facing the prospect of changing jobs and tasks in the future, may be those that have more to lose from automation, precisely because they are the individuals who have invested more in their human capital formation, and they have therefore higher expectations about the working conditions that they feel they deserve.

**Proposition 2:** Fear of replacement will negatively affect the job satisfaction of low-skilled workers more than that of high-skilled workers if the former effect is stronger than the latter.

## 4. Data

### Individual-level data

We use the *Working Life Barometer* survey (*Arbeidslivsbarometer*), which provides annual microdata for several thousand Norwegian workers. The survey is provided by the Confederation of Vocational Unions (YS), a politically independent umbrella organization for labor unions, and organized by the Work Research Institute in Norway. TNS Gallup collects the data targeting a large random sample of Norwegian workers aged 18–67 years. Our analysis makes use of the four surveys carried out in the years 2016 to 2019, which include information on the main variables of interest for this study, and particularly workers’ subjective assessments of the threats of automation, and their job satisfaction.

The main target variable in the study is job satisfaction, which is measured by means of responses to the survey question: “*How satisfied are you with your job*?*”*. Respondents indicate their satisfaction level on a 1–5 scale (“Very dissatisfied”; “Pretty dissatisfied”; “Neither satisfied nor dissatisfied”; “Pretty satisfied”; “Very satisfied”). The main explanatory variable is fear of replacement. This is measured by means of responses to the following survey question: “*Do you think some of your current tasks could be done by machine instead*?”. Fear of replacement is a dummy variable: respondents who answer yes to this question take value 1, whereas workers who do not think that their tasks could be replaced by a machine take value 0. It is important to observe that this survey question measures workers’ assessment of the possibility that their tasks could be replaced by machines (cognitive reaction), and not directly the fear to lose their job as a consequence of automation (emotional reaction). However, as we will show later in the results section, this survey question is closely related to other survey questions that measure workers’ fear of losing their job, and it is therefore reasonable to use it as a proxy measure of fear of replacement. It is also worthwhile to note that only workers who are currently employed are asked to answer the question on fear of replacement, whereas unemployed individuals must skip this part of the questionnaire. Hence, our analysis focuses on the beliefs of workers who are potentially exposed to automation, but it does not consider those individuals that have already been laid off due to automation.

Next, another important variable in our study is the skill-level of workers, which is measured by their education level, distinguishing workers with a completed University degree *versus* those without tertiary education. In terms of control variables, the *Working Life Barometer* survey also provides employee-level demographic and socio-economic information such as age, gender, income, union membership, and occupation type. In total, we analyze responses from 10,051 workers aged 19–68 years. Table 1 presents a list of the variables used in the analysis, and Table 2 reports some descriptive statistics.

**Table 1:** Variables.

Variable	Definition
	*Individual level variables*
Job satisfaction	Respondents indicate their job satisfaction ranging from 1 “Very dissatisfied”; 2 “Pretty dissatisfied”; 3 “Neither satisfied nor dissatisfied”; 4 “Pretty satisfied”; 5 “Very satisfied”.
Machine replacement	Respondents indicate whether they believe that a machine can perform some of their job tasks.
Union membership	Dummy indicating whether the respondent is unionized.
Age	Age of respondent.
Women	Dummy indicating the gender of the respondent.
University degree	Dummy indicating whether the respondent has a university degree.
Working in industry	Dummy indicating whether the respondent is an industry worker.
	*Regional level variables*
ΔRobot exposure	Industry-region’s long-term robot adoption per thousand workers. More detailed definition in main text.
Unemployment benefit recipients	Share of regional population that are registered recipients of unemployment benefits.
Business building broadband infrastructure availability	Fixed broadband penetration per 100 inhabitants.
Population	Log of regional population.
GDP per capita	Log of regional GDP per capita.
Tertiary education	Regional share of population (aged 25–64) with tertiary education.
Share of big industrial companies	Big industrial companies as share of total firm population by region.

**Table 2:** Descriptive statistics.

Variable	Obs.	Mean	Std. Dev.	Min	Max
Job satisfaction*	10,051	3.99	0.84	1.00	5.00
Machine replacement	10,051	0.40	0.49	0.00	1.00
Union membership	10,051	0.69	0.46	0.00	1.00
Age	10,051	46.44	11.67	19.00	68.00
Income scale	10,051	4.81	1.81	1.00	9.00
Women	10,051	0.52	0.50	0.00	1.00
University degree	10,051	0.56	0.50	0.00	1.00
Working in industry	10,051	0.08	0.26	0.00	1.00
Robot exposure	10,051	0.06	0.03	0.00	0.20
Log(GDP per capita)	10,051	12.89	0.53	12.14	13.69
Tertiary education (share of population)	10,051	43.81	6.68	35.5	54.3
Business building broadband infrastructure availability	10,051	0.74	0.14	0.56	0.97
Log(population)	10,051	14.09	0.20	13.74	14.33
Unemployment benefit recipients (share of population)	10,051	4.34	0.45	3.41	5.12
Share of big industrial companies	10,051	10.72	1.20	8.05	12.61

* Job satisfaction: Very dissatisfied: 1.17%; pretty dissatisfied: 4.98%; neither satisfied nor dissatisfied: 13.57%; pretty satisfied: 53.86%: Very satisfied: 26.42%.

### Robot data

To measure the introduction of industrial robots in local labor markets in Norway, we make use of a dataset provided by the International Federation of Robotics (IFR), which contains information on robot stock and deliveries in Norwegian industrial firms since 1993. The IFR defines an industrial robot as an “*automatically controlled*, *reprogrammable multipurpose [stationary or mobile machine]*” [32]. Following this definition, industrial robots are autonomous machines capable of operating without human intervention and that could potentially substitute or complement human labor. The IFR provides detailed data on robot stock and deliveries for the period 1993–2017, which can be broken down by application or industry. Robot stock for years 2018 and 2019 are extrapolated assuming a 9 percent annual growth in operational stock as projected by IFR [33]. IFR data have recently been used to analyze the impact of automation on employment and wages [22,34,35], as well as on workers’ well-being [11,12].

We allocate robots in regional labor markets following extant research [22,34,36], assuming that robots are distributed across region and industries by their respective employment shares. Employment shares are calculated based on Eurostat’s Labor Force Survey data dating back to 2008. The long-term change in robot adoption occurs between years 2008 and *t* based on initial regional employment composition in each industrial category (industry, agriculture, construction, and services), with the change in robot adoption per 1,000 workers fixed at the starting level in year 2008.

$$\Delta {robot\;exposure}_{r,s,t}=\sum_{s\in S}\frac{{emp}_{r,s,2008}}{{emp}_{r,2008}}*\left(\frac{{robots}_{s,t}-{robots}_{s,2008}}{{emp}_{s,2007}}\right)$$ (1)

In this setup, robot exposure is measured as national robot adoption allocated at the region-industry level (*r*,*s)*. Each regional labor market *r* is scaled by the nation’s total employment *emp*_*c*_. In short, the instrumental variable that we will use in our empirical analysis is the long-term change in the adoption of industrial robots by Norwegian firms in each local labor market (i.e. in each region-industry *r*,*s)*. This measures the extent to which workers have been exposed to automation from 2007 onwards (see a further discussion of the empirical identification strategy in section 3.2 below).

### Regional-level control variables

We use the Eurostat’s Labor Force Survey to obtain regional-level variables on GDP per capita, population share with tertiary education, and population size. From Statistics Norway, we retrieve data on firms by size for each region. Further, we collect data on unemployment benefit recipients as a share of total population from the Norwegian Labour and Welfare Administration (NAV), for each region and each year of our dataset.

To avoid omitting the possible conflating influence of ICTs when analyzing automation, previous studies have included ICT capital or investment as an additional control variable [34,37]. However, others argue that more specific measures of ICT utilization are necessary for micro-level studies [38]. Unlike existing studies that have analyzed the impact of high-speed broadband developments in Norway [39,40], we use as additional control variable the broadband internet availability in office buildings instead of households in each region. Data on office buildings with at least 8/8 Mbit/s speeds are provided by the Norwegian Communications Authority (Nkom), and matched against individuals through regional identifiers.

## 5. Empirical methods

The econometric analysis sets out to study the relationship between fear of replacement and job satisfaction. Fear of replacement is the subjective assessment that each worker does on the possibility that her working tasks will be replaced by a smart machine in the future. Such subjective assessment may arguably depend on unobserved and idiosyncratic factors such as e.g. ability, attitude towards risk, and technological/digital competencies. Therefore, unobserved individual factors might possibly influence both the outcome variable (job satisfaction) and the main explanatory variable (fear of replacement).

To address endogeneity concerns, we follow recent research and use the lagged introduction of robots in local labor markets (industry-regions) as an instrument for individual workers’ fear of replacement [11,12]. Existing studies on robot implications for labor markets where robot adoption is the main explanatory variable address endogeneity issues by incorporating spillover effects from robot adoption across industries in other countries as an instrument in a 2SLS setup [22,34,36]. Unlike these studies, we approach subjective responses to structural inroads of robot technology in local labor markets to identify learning effects from past automation. Specifically, our instrumental variable is the one defined in (1) above, i.e. the change in the adoption of industrial robots by Norwegian firms in each local labor market (industry-region) between 2008 and year *t* (i.e. one of the survey years 2016–2019). This variable measures the extent to which workers in each of the 16 industry-regions considered in this study have been *exposed* to rising automation in recent years. We thus exploit (lagged) variation in robot adoption over time and across industry-regions in Norway to instrument for individual fear of replacement at time *t*. The underlying idea of this identification strategy is that workers that are employed in local labor markets that have more rapidly been exposed to automation (i.e. in industry-regions where firms have increasingly used industrial robots) will be more likely to consider automation as a possible threat, and therefore fear that some of their working tasks could be replaced by a machine in the future. In other words, we posit that workers learn from past robot adoption in their local labor markets, because they are subject to *peer effects* [41]. Although it is reasonable to posit that these peer effects work through *changes* in robot adoption over time, we cannot exclude the possibility that the same mechanism may also work through the absolute *levels* of robot adoption (i.e. workers may fear replacement when they experience a high intensity of industrial robots in the industry-region where they work). To consider this possibility, we have also calculated our instrumental variable in levels rather than as changes over time, and reported additional regressions in the online appendix (see Table A5 in S1 File, whose results are in line with the main results presented in the paper).

**Fig 1:**
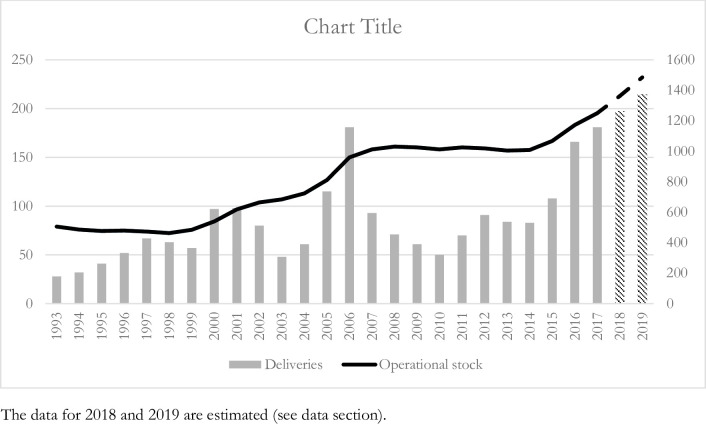
Robot deliveries and operational stock for Norway between 1993 and 2019. The data for 2018 and 2019 are estimated (see data section).

**Fig 2:**
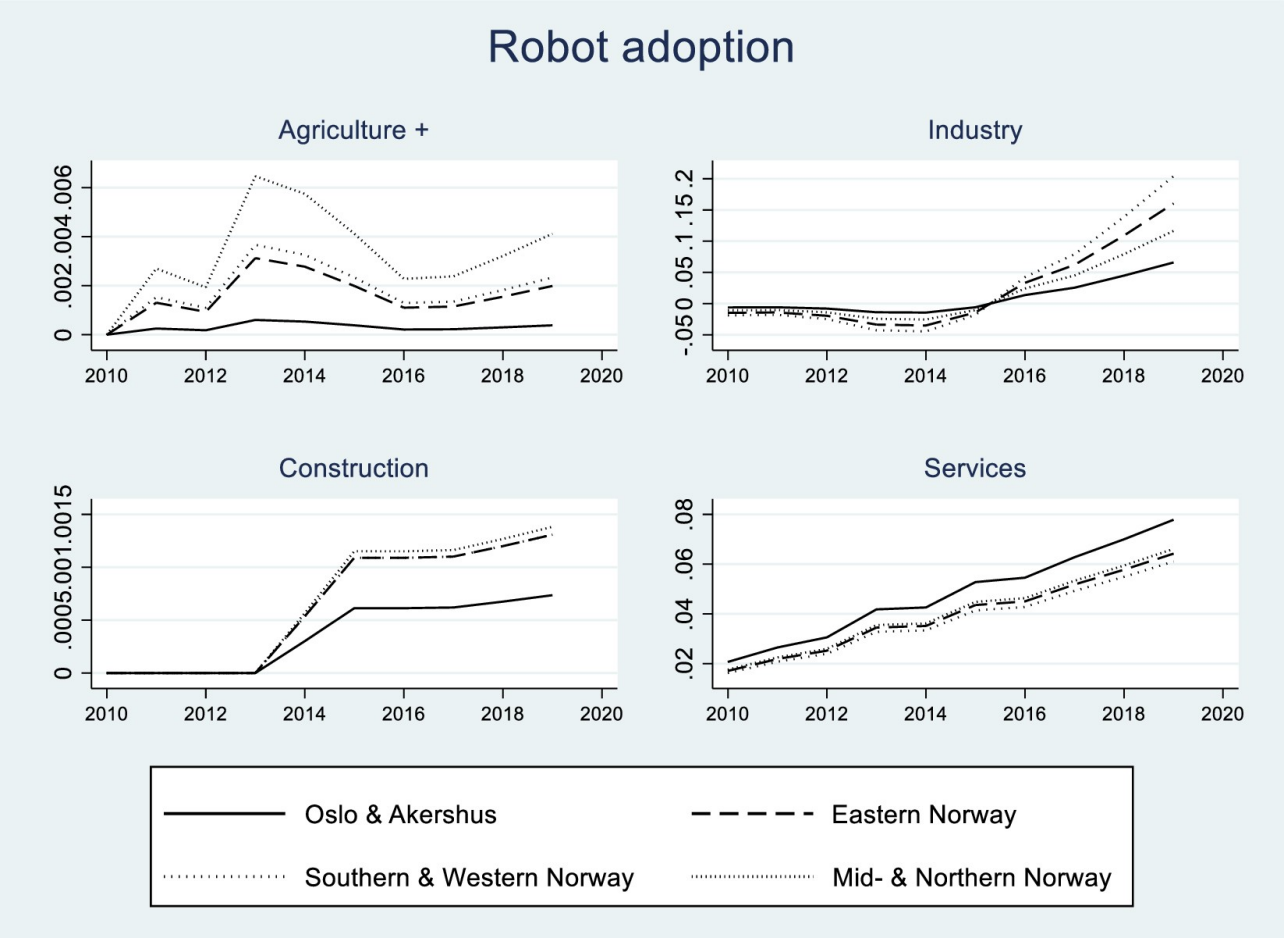
Robot adoption by industry-region, 2010–2019.

Norwegian firms have invested in sophisticated robotics and automation technologies to keep pace with the Digital Single Market strategy [42], and our empirical analysis exploits this exogenous source of tempo-spatial variations to identify the effects of automation on workers’ job satisfaction. Fig 1 illustrates the dynamics of industrial robots adoption in Norway in the last decades, showing a much faster pace since 2014. Table 3 shows that most robots have so far been used by firms within manufacturing, and less so in other branches such as agriculture, construction and services. However, Table 3 also shows that the introduction of robots by service firms has been quite rapid in the last decade. Fig 2 illustrates the trend in robot adoption since 2010, indicating a rising trend in all 16 industry-regions considered in this study, and particularly so in manufacturing and services.

**Table 3:** Adoption of robots (operational stock) in Norwegian regions and industries.

Region	Sector	2008	2017
Oslo & Akershus	Agriculture, forestry and fishing	0	1
	Industry	113	140
	Construction	0	0
	Services	3	13
Eastern Norway	Agriculture, forestry and fishing	3	4
	Industry	296	294
	Construction	0	1
	Services	3	11
Southern & Western Norway	Agriculture, forestry and fishing	3	4
	Industry	444	521
	Construction	0	1
	Services	3	14
Mid- and Northern Norway	Agriculture, forestry and fishing	3	5
	Industry	141	173
	Construction	0	0
	Services	3	13

To get a further overview of the diffusion and use of industrial robots in Norway, it is also useful to get some descriptive figures from Eurostat’ survey on “ICT usage and e-commerce in enterprises (2018)” (see Tables A1 to A4 in S1 File). Large firms are the main adopters of both industrial and service robot technologies, and capital-intensive firms appear to invest in and integrate both technologies in their operations. Operating machines represent about 60% of all industrial robots in Norwegian firms in 2017. Whereas large firms use service robots for mostly logistics and transportation purposes, small and medium enterprises (SMEs) deploy robots in more product-related purposes, such as inspection, assembly or construction works.

Although our paper focuses on industrial automation, workers in knowledge-intensive service occupations may rather fear competition from new artificial intelligence technologies. Table A4 in S1 File presents some descriptives on Norwegian firms’ use of Big Data in their business operations. Large firms are more likely to use Big Data than SMEs. Large firms use smart sensors (e.g. Internet of Things) and geo-data to a greater extent than SMEs. On the other hand, SMEs more actively collect data from social media for marketing purposes. In sum, smart machines are swiftly making inroads in the Norwegian economy, and this pace has accelerated in the last five years.

**Fig 3:**
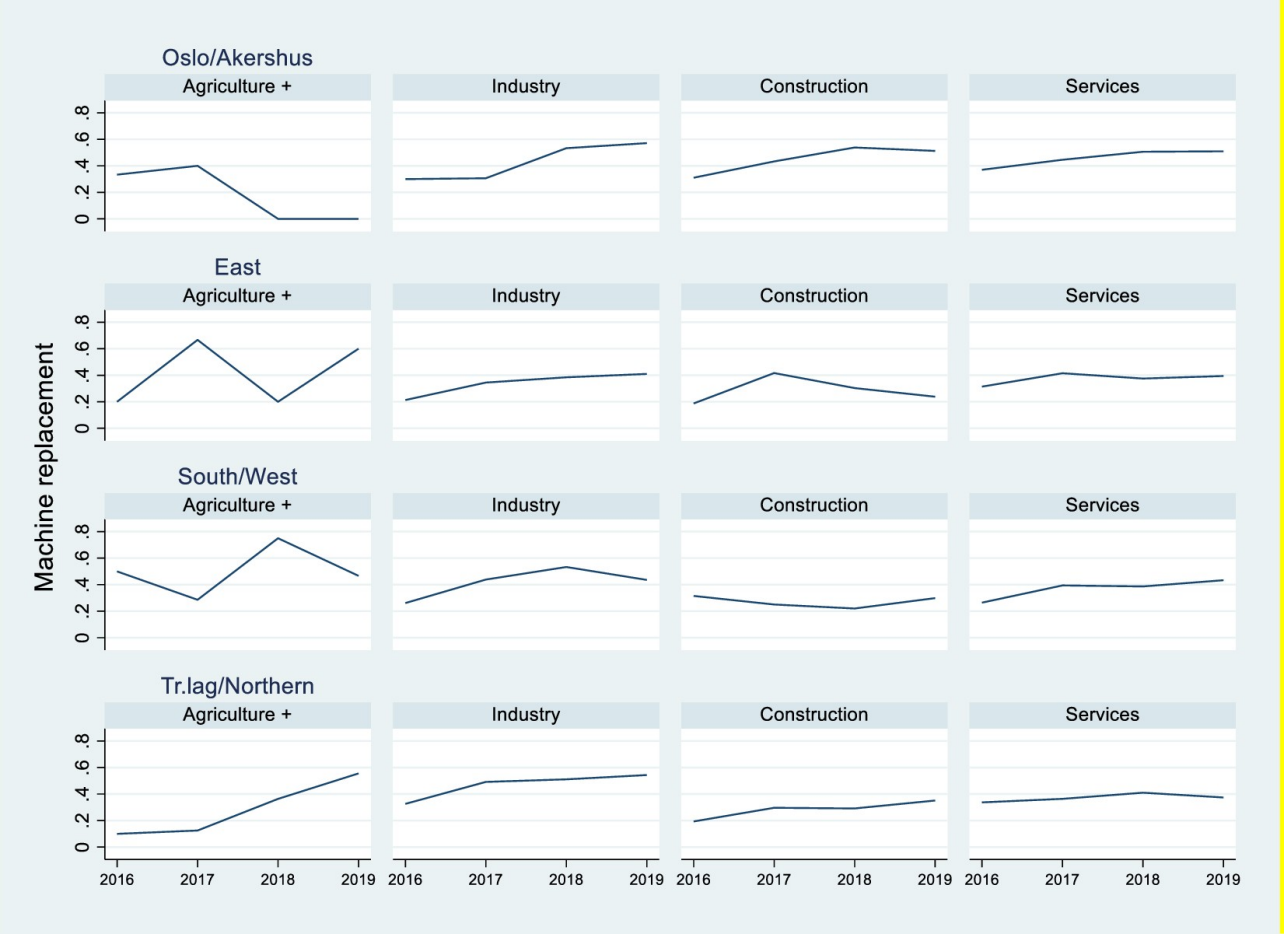
Share of workers who believe that their job can be replaced by machines, by region and industry.

Fig 3 shows the time trend of the variable machine replacement for each of the 16 industry-regions in the more recent period 2016–2019 to which our survey data refers. Although this is a relatively short span (which does not make it possible to assess long run trends), Fig 3 indicates that fear of replacement due to automation has increased steadily in most of the industry-regions considered in this study, and that there is by and large a correspondence between the time trends reported in Fig 2 (robot adoption) and Fig 3 (machine replacement).

Based on the identification strategy noted above, we estimate a two-stage instrumental variables (IV) model: the first stage (3) investigates how robot exposure and other control factors explain variations in workers’ fear of replacement, whereas the second stage (2) estimates the relationship between job satisfaction and anticipated replacement:

$${JS}_{irst}=\alpha_{1}+\gamma {machine\;replacement}_{irst}+\delta x_{irst}^{'}+\eta_{r}+\theta_{t}+\varepsilon_{irst}$$ (2)

$${machine\;replacement}_{irst}=\alpha_{2}+\mu z_{rst}+\rho x_{irst}^{'}+\tau_{r}+\varphi_{t}+\in_{irst}$$ (3)

*JS* is reported job satisfaction, *machine replacement* is the dummy variable indicating whether the respondent believes a machine can perform her/his job tasks, *z* is the instrumental variable (industry-region lagged pace of robot adoption), and *x* is a set of covariates (measured for individuals in each survey wave). The subscript *r* denotes the geographical region of residence of each worker *i*, *s* denotes the industry in which the worker is employed, and the subscript *t* refers to survey year. Among the set of covariates, the skill variable is particularly relevant for the present study, as we seek to investigate whether the relationship between fear of replacement and job satisfaction differs for high- *versus* low-skilled workers. To test these moderation effects, we interact the skill variable with the robots variable in the first stage equation, and with the fear of replacement variable in the second stage equation.

For model identification, the vector *x* in Eqs (2) and (3) does also include detailed demographic and socio-economic characteristics expected to correlate with job satisfaction and anticipated replacement, such as age, gender, income, union membership, and industry employment. According to previous studies, these factors are relevant to explain variation in job satisfaction, labor dynamics and technological automation diffusion [8,22,31,34,37,43–45]. Finally, both equations also include a full set of regional dummies and time dummies that control for unobservable determinants of job satisfaction within each region over time.

It is important to note that our identification strategy is based on the assumption that robot exposure in each industry-region will affect workers’ job satisfaction only through its effects on fear of replacement, and we therefore exclude a direct impact of robot exposure on job satisfaction. Conceptually, we cannot exclude that robot adoption in a given firm may potentially affect employees’ job satisfaction directly, and not only indirectly through fear of replacement. However, we think that this conceptual argument is not a particular reason of concern in our empirical study. The reason for this is that robot adoption in Norway, although it has increased rapidly during the last few years, it is still relatively low in absolute levels (around 6%, see Table 2). This means that our dataset and estimations do not refer to workers who already use robots in their current job, but for the great majority they refer to workers that are exposed to (i.e. observe) automation being introduced in other firms in the industry-region where they work, and that due to these peer effects fear that machines could replace some of their working tasks in the future.

The econometric model is estimated as a two-stage bivariate recursive ordered probit maximum likelihood setup, which accommodates the ordinal character of the outcome and main explanatory variable [46,47]. This model estimates response probabilities of two variables, one ordered and one dichotomous, and the exogenous variable robot exposure is included in the first stage [48,49]. Estimations are performed with Roodman’s [50] conditional mixed process (CMP) program. Because the instrument is measured at the industry-region level, estimations are likely to contain grouped structures, and we therefore cluster standard errors in all regressions [41,51,52].

## 6. Results

### First stage results

Table 4 presents the estimation results of the first stage (Eq 3), in which the dependent variable is machine replacement (i.e. workers’ self-reported assessment of the possibility that some of their working tasks will be replaced by a smart machine in the future). Table 4 reports estimation results for both the model without control variables and the one including the full set of controls, in order to assess whether the inclusion of controls affect the results [53,54]. The results for the two models are however very close to each other. We begin by briefly looking at the results for the set of control variables, before turning attention to the main variables of our interest. Among the controls, Table 4 shows that fear of replacement is stronger for younger workers. These have a longer time horizon remaining in their working life, and they are therefore more likely to expect that automation will replace some of their working tasks in the future [12]. Employees that belong to a trade union are less likely to fear replacement, arguably because their employment and working conditions are partly protected by the trade union membership (we elaborate further on this in section 6 below). Regarding wage levels, fear of replacement is stronger for workers that have higher income. A possible interpretation of this finding is that, after controlling for education and skill levels (that are correlated with wages and that also affect fear of replacement), workers with higher income have more to lose *vis-à-vis* workers with lower income, since automation of tasks may lead, in absolute terms, to a stronger wage decrease for them. Further, we control for gender and sector of occupation (industry employment), which are two standard control variables in studies of workers’ subjective well-being.

**Table 4:** First stage results. Dependent variable: Machine replacement.

	(1)	(2)	(3)	(4)
Robot adoption	0.711***	0.853***	1.845***	2.250***
	(0.217)	(0.256)	(0.570)	(0.681)
Age		-0.004***		-0.011***
		(0.001)		(0.002)
Union membership		-0.056***		-0.149***
		(0.013)		(0.035)
Income scale = 2		-0.093***		-0.268***
		(0.024)		(0.065)
Income scale = 3		-0.006		-0.019
		(0.022)		(0.057)
Income scale = 4		0.024		0.063
		(0.023)		(0.060)
Income scale = 5		0.027		0.072
		(0.029)		(0.077)
Income scale = 6		0.043		0.112
		(0.034)		(0.090)
Income scale = 7		0.078***		0.206***
		(0.026)		(0.069)
Income scale = 8		0.039		0.100
		(0.026)		(0.070)
Income scale = 9		0.069		0.182
		(0.053)		(0.137)
University degree		0.037*		0.098*
		(0.021)		(0.055)
Woman		-0.011		-0.030
		(0.017)		(0.044)
Industry employment		-0.044**		-0.119***
		(0.016)		(0.042)
Controls				
Regional dummies	✓	✓	✓	✓
Year dummies	✓	✓	✓	✓
F-stat	10.77	11.15		
N	10,051	10,051	10,051	10,051

Robust standard errors in parentheses are clustered for workers in the same region and industry. Columns 1 and 2 present OLS estimates. Columns 3 and 4 show probit estimates.

* p<0.10,

** p<0.05,

***p<0.01.

Shifting the focus to the main variables of interest for this study, the instrumental variable (changes in robot adoption in local labor markets between 2008 and year *t*) is as expected positively correlated with the dependent variable (workers’ fear of replacement). As explained in section 5, the underlying idea is that when individuals work in industry-regions in which firms have increasingly been using robots in the last few years, they are more *exposed* to automation (e.g. because some of their peers or acquaintances in the same region have lost their job due to automation). These peer effects translate into fear of replacement even for workers that are still employed and not directly touched by automation technologies yet. Table A6 in S1 File corroborates this interpretation by presenting first stage regressions in which we use two additional control variables that measure *fear of job loss*: (1) job loss worry (“To what extent are you worried about losing your job?”); (2) unemployed in five years (“Do you expect to be unemployed within the next five years?”). Both of these control variables are positive and significant in the regressions, indicating that fear of job loss (emotional reaction) and machine replacement (cognitive reaction) are closely related dimensions. Further, the inclusion of these additional control variables does not affect the size and significance of the estimated effect of the instrumental variable robot adoption on machine replacement.

What is the economic significance of these results? According to our OLS estimates (see column 2 in Table 4), a one standard deviation increase in robot exposure increases the probability that a worker expresses fear of machine replacement by 2.6 percent (we thank an anonymous reviewer for suggesting to point this out). It is hard to say whether this estimated effect is economically sizeable. However, considering that robot adoption in Norway has more than doubled during the time span considered in this study, we may think that the overall effect of automation on fear of replacement has arguably been important during this period. It is also interesting to assess this figure in the light of the effects of robot adoption on employment found in other recent studies (although none of these present estimates of the effects of robot adoption on subjective fear of replacement). [34] find that one additional robot per thousand workers reduces the employment rate by 0.16–0.20 percentage points across European regions. In their study of the U.S. labor market, [22] find that one robot reduces employment by three workers. Further, [36] suggest that the corresponding number for German manufacturing workers is about two jobs. However, the overall effect on German employment is unchanged as the job reduction in manufacturing is offset by gains in the service sector.

Next, we look at the results for the other important variable considered in this study: workers’ skills. Table 4 shows that individuals with tertiary education have on average a greater fear that some of their working tasks could be replaced by a machine in the future. As discussed in section 3, this might be explained by the fact that high-skilled workers are typically more educated individuals who read more and follow societal debates on the media about robots, AI and automation, and their negative consequences for employment. Hence, high skilled workers are arguably more exposed to peer effects, which may translate in a greater fear about the future of employment.

Relatedly, how do workers’ skills affect the positive relationship between automation and fear of replacement? To test this moderation effect, Table 5 reports estimation results of the first stage equation by workers’ skill level. While the estimated coefficient of the robot adoption variable is positive and significant for both workers with tertiary education and those without a college degree, the size of this effect is larger for the latter group. This moderation effect is in line with the recent literature on the effects of automation on employment, which shows that low-skilled workers are more exposed to the risks of displacement from automation because they typically carry out routine tasks that can more easily be automated [3,4,16,20].

**Table 5:** First stage results by workers’ skill level.

	(1)	(2)
	No university education	University education
Robot adoption	1.040***	0.749**
	(0.306)	(0.283)
Age	-0.004***	-0.004***
	(0.001)	(0.001)
Union membership	-0.024	-0.085***
	(0.020)	(0.020)
Income scale = 2	-0.111**	-0.048*
	(0.042)	(0.023)
Income scale = 3	0.005	-0.024
	(0.052)	(0.036)
Income scale = 4	0.037	0.031
	(0.050)	(0.018)
Income scale = 5	0.092	0.010
	(0.070)	(0.024)
Income scale = 6	0.024	0.067*
	(0.047)	(0.034)
Income scale = 7	0.099	0.080**
	(0.066)	(0.033)
Income scale = 8	0.061	0.032
	(0.051)	(0.035)
Income scale = 9	0.072	0.068
	(0.056)	(0.062)
Woman	0.052**	-0.058***
	(0.023)	(0.018)
Industry employment	-0.057***	-0.005
	(0.018)	(0.030)
Controls		
Regional dummies	✓	✓
Year dummies	✓	✓
N	4,434	5,617

Robust standard errors in parentheses are clustered for workers in the same region and industry. Columns 1 and 2 present OLS estimates.

* p<0.10,

** p<0.05,

*** p<0.01.

### Second stage results

Table 6 reports estimation results for the second stage of the model (Eq 2), in which job satisfaction is the dependent variable. The table reports first the results for the model without control variables and then those for the model including the full set of controls. The results for the two models are very close to each other, indicating that the inclusion of controls does not affect the results on the main explanatory variables [53,54]. The control variables that we use are commonly used in the job satisfaction literature. Income is positively correlated with job satisfaction, in line with extant literature showing that wage is one of the factors that enhance workers’ subjective well-being [7,8]. Female workers report on average higher job satisfaction than males; and individuals employed in manufacturing (industry) have lower satisfaction than average, a finding that is explained in the subjective well-being literature by the fact that factory workers typically carry out routine and monotonous working tasks and have a lower degree of autonomy and creativity [25].

**Table 6:** Second stage results. Dependent variable: Job satisfaction.

	(1)	(2)	(3)	(4)
Machine replacement	-1.268**	-0.760**	-1.093***	-0.999***
	(0.512)	(0.304)	(0.117)	(0.169)
Age		0.007***		0.008***
		(0.001)		(0.002)
Union membership		-0.041*		-0.057***
		(0.024)		(0.020)
Income scale = 2		-0.135**		-0.137***
		(0.067)		(0.052)
Income scale = 3		0.009		0.016
		(0.037)		(0.038)
Income scale = 4		0.093*		0.120**
		(0.053)		(0.054)
Income scale = 5		0.144***		0.187***
		(0.044)		(0.049)
Income scale = 6		0.195***		0.246***
		(0.057)		(0.066)
Income scale = 7		0.214***		0.285***
		(0.049)		(0.053)
Income scale = 8		0.203***		0.274***
		(0.060)		(0.073)
Income scale = 9		0.322***		0.434***
		(0.071)		(0.094)
University degree		0.035		0.034
		(0.024)		(0.028)
Woman		0.092***		0.129***
		(0.020)		(0.028)
Industry employment		-0.169***		-0.213***
		(0.020)		(0.026)
Controls				
Regional dummies	✓	✓	✓	✓
Year dummies	✓	✓	✓	✓
N	10,051	10,051	10,051	10,051

Robust standard errors in parentheses are clustered for workers in the same region and industry. Columns 1 and 2 present 2SLS linear estimates. Columns 3 and 4 show bivariate recursive probit estimates.

* p<0.10,

** p<0.05,

***p<0.01.

The main variable of interest in Table 6 is machine replacement. The estimated coefficient for this variable is as expected negative and significant. This means that workers that report higher fear of replacement from smart machines have on average lower job satisfaction. As noted in section 3, the reason for this is that for workers that are currently employed, the prospect that smart machines could replace some of their current working tasks in the future, or even the whole job, does create uncertainty about future job prospects and personal finance, thus lowering job satisfaction. The marginal effects for each category of the job satisfaction variable (not reported in Table 6) indicate that a change in the fear of replacement dummy variable (i.e. from “no fear” to “fear of replacement”) decreases the job satisfaction of the “very satisfied” workers by around 30%, and of the “very or pretty dissatisfied” workers by around 6–11%.

Table A7 in S1 File reports second stage regressions that also include two additional control variables that measure *fear of job loss*: (job loss worry; unemployed in five years; see definition of these two survey questions in section 6 above). The additional control variables are positive and significant in the regressions, and their inclusion in the model does not affect the size and significance of the estimated effect of machine replacement on job satisfaction, indicating that fear of replacement due to automation is important for workers’ subjective well-being even after controlling for the more general construct fear of job loss.

How is this relationship moderated by workers’ skill level? Table 7 investigates this question by reporting marginal effects of the machine replacement variable for workers that have tertiary education *versus* those that do not have a college degree. The table shows that the marginal effect for the workers without a college degree is negative and significant, indicating that fear of replacement increases the probability that low-educated workers will report high job satisfaction by nearly 50%. On the other hand, the corresponding marginal effect for the workers with a University degree is not statistically significant. Abeliansky and Beulmann [11] also carried out some regressions to study the relationships between automation and mental health for different educational groups (tertiary vs secondary education), but they did not find any significant difference among these groups of workers in Germany. As discussed in section 3, the interpretation of our finding is that low-skilled workers, if replaced, will on average have fewer possibilities to find another occupation in the labor market. This is in line with recent literature that provides evidence that displacement effects of industrial robots on employment and wages are stronger and more significant for low-education workers [20,22]. On the other hand, automation technologies can have more positive effects on high-skilled workers, increasing the demand for labor, wages and the complexity and interest of their tasks [18]. In short, we posit that workers are at least to some extent aware of the distinct impacts that automation can have for different types of occupations, and this explains why fear of replacement turns out to be a concern for low-skilled employees.

**Table 7:** Second stage results by workers’ skill level (marginal effects of machine replacement for workers of different education levels).

	Below university education	University education
Machine replacement	-0.494***	0.295
	(0.165)	(0.683)
Controls		
Individual controls	✓	✓
Regional dummies	✓	✓
Year dummies	✓	✓
N	10,051	10,051

Robust standard errors in parentheses are clustered for workers in the same region and industry. Columns 1 and 2 present results from bivariate recursive probit estimates.

* p<0.10,

** p<0.05,

*** p<0.01.

It may be argued that the education level dummy variables that we have used in these regressions only reflect formal education acquired through the school and University system, and disregards other skills that workers acquire during the working life through training, apprenticeships and learning by doing. Ideally, if we had information about each worker’s occupation, we could construct a proxy measure for skills by using the three-level job complexity schema developed by Hunter et. al. [55], which creates a correspondence between job types and corresponding skill content. However, our dataset does not have information about workers’ occupation type, and we are therefore not able to follow this route. Hence, in the absence of a more specific variable measuring workers’ skills, we carry out two additional exercises. First, we use age as an additional proxy of workers’ skills and abilities to perform their job. Table 8 reports marginal effects of machine replacement on job satisfaction for workers of different education levels *and* for different age groups. The results confirm the main finding noted above. The marginal effect of anticipated machine replacement on present job satisfaction of the workers without a college degree is negative and significant for all sub-groups (except those younger than 30), and it is not significant for workers with a University degree. This corroborates the main finding of our analysis that machine replacement has a negative effect on job satisfaction, and that this effect is particularly relevant for low-skilled workers.

**Table 8:** Second stage results by workers’ skill level and age (marginal effects of machine replacement for workers of different education levels and different age groups).

	Below university education	University education
Age group		
<30 years	0.109	1.552
	(0.363)	(2.205)
30–44 years	-0.427**	-1.306
	(0.171)	(0.871)
45–59 years	-0.385*	0.138
	(0.205)	(0.443)
60+ years	-1.180***	-0.486
	(0.235)	(1.478)
Controls		
Individual controls	✓	✓
Regional dummies	✓	✓
Year dummies	✓	✓
N	10,051	10,051

Robust standard errors in parentheses are clustered for workers in the same region and industry. Columns 1 and 2 present results from bivariate recursive probit estimates.

* p<0.10,

** p<0.05,

***p<0.01.

Second, it may be argued that the education variable does not only measure workers’ skills, but it is also a proxy for *employability*, since workers with higher education levels can more easily find a new job. If so, employability, rather than skills, could be the latent variable moderating the effect of fear of replacement on job satisfaction. To address this possibility, we make use of two additional variables measuring employability: (1) Difficult to find a new job (“How difficult or easy do you think it would be for you to find a job that is at least as good as the one you have now?”); (2) Insufficient skills in current job (“How often do you experience insufficient competence to perform your tasks?”). Then we include these two variables as additional controls in our first and second stage regressions, and report the results of these robustness tests in tables A8 to A10 in S1 File. First stage results (Tables A8 and A9 in S1 File) show that the inclusion of the additional controls for employability does not affect the main result about the effect of robot adoption on machine replacement, and that this effect is still stronger for workers with lower education level. Second stage results (Table A10 in S1 File) are also in line with our baseline estimations: in the extended model specification that controls for employability, the effect of machine replacement on job satisfaction is still negative and significantfor workers of lower education level (across age groups). In short, these additional exercises show that, even when we control for employability, workers’ education level moderates the effect of fear of replacement on job satisfaction, and it may thus be considered as a reasonable proxy measure of latent workers’ skills.

### Robustness tests

Our identification strategy rests on the assumption that the (lagged) introduction of robots in local labor markets in Norway affects current job satisfaction only through its effects on workers’ fear of replacement. Although our regressions control for a set of relevant employee-level characteristics and include region- and time fixed effects, it is also useful to carry out some additional robustness exercises to test the potential confounding effects of omitted variables that may in principle affect both fear of replacement and the error term of the outcome equation.

Tables A11 and A12 in S1 File report estimation results of first and second stage regressions that include some additional region-level control variables in the model. The first two columns add region’s GDP and tertiary education level, which may be thought to be general relevant factors that may drive both the introduction of industrial robots and job satisfaction patterns. Though, the estimated coefficients of the instrumental variable robot adoption (Table A11 in S1 File) and of the machine replacement variable (Table A12 in S1 File) are still significant and stable after the introduction of these two possible confounding factors. Regressions in column 3 add a variable measuring business building broadband infrastructure in each region. The reason for including this variable is that ICT diffusion may be a potentially conflating factor that can disturb the effect of robots adoption on employment [3,37]. By controlling for broadband internet access in office buildings we address this concern, reasonably assuming that the development of broadband infrastructure is driven by policies and investments that are exogenous to the individual worker. Again, the inclusion of this additional control does not affect the estimated coefficient of the robot variable in Table A11 in S1 File, and of the machine replacement variable in Table A12 in S1 File. These coefficients still have the same signs and significance levels, and their estimated size is slightly larger than in baseline regressions. Finally, columns 4, 5 and 6 also add three other region-level controls: unemployment benefit recipients (share of population in each region), share of large companies in each region, and population size (log). The unemployment benefit variable controls for the possible confounding effect of different unemployment rates across regions. The share of large companies takes into account the fact that large firms do on average have a higher rate of introduction and use of industrial robots (see Tables A1 and A4 in S1 File), so that employees in regions with a high share of large firms are potentially more exposed to the effects of automation. Finally, the population variable is a standard control for the size and density of the region, which may be related to the extent and intensity of peer effects that affect workers’ fear of replacement. However, the inclusion of these additional variables does not affect the main results for the explanatory variables of our interest.

As a further robustness test, Table A13 in S1 File reports the results of a placebo test that adds a lead variable–robust exposure at year *t+1* –to the set of regressors in the first stage equation (including also the three additional region-level control variables noted in the previous paragraph). In these placebo regressions, the future robot adoption variable is not significant, and its inclusion does not affect the sign and size of the estimated coefficient of the instrumental variable (lagged pace of robot adoption). This further rules out the possibility that our results are driven by some omitted variables that are related to both job satisfaction and robot adoption.

Next, it is interesting to consider the question on whether our instrumental variable (pace of robot adoption in industry-regions) should be regarded as a *peer effect* or rather a *neighbor effect*. Table A14 (in S1 File) considers this question by including two additional control variables in the first stage regressions. The first additional control is robot adoption in the other three industries in the same region; and the second one is robot adoption in the same industry in all other regions in Norway. In other words, these two additional controls are proxies for inter-industry and inter-regional neighboring effects, respectively. Table A14 in S1 File shows that none of these variables are significantly correlated with fear of replacement, and their inclusion does not affect the precision of the instrumental variable (robot adoption in a given industry-region). These robustness exercises provide further evidence that our instrumental variable catches peer effects that are specific to each industry-region, and that hold even after controlling for neighbor effects related to robot adoption in surrounding regions and industries in Norway.

Finally, it is relevant to comment further on the role of one of the control variables in the model: union membership. As noted in relation to Table 4 (and other first stage results reported in the online appendix), workers that belong to a trade union do on average report lower fear of machine replacement. This may suggest that workers in trade unions feel they are more protected from the impacts of industrial robots. However, this pattern is in contrast with Acemoglu and Restrepo [44], which find a positive association between industrial robot adoption and unionization rates across countries, arguing that this is due to the fact that unionization may raise labor costs. Yet, skill-biased technical change also creates a stronger incentive for deunionization because the outside employment and wage options of skilled workers have improved [56]. To investigate this further, we run additional regressions in which we interact our two main explanatory variables (robot adoption in the first stage, and machine replacement in the second stage) with the union membership variable. The idea is to test directly whether Norwegian workers that belong to a trade union do on average think that they are less likely to be affected by automation. However, the results of these regressions (reported in Table A14 in S1 File) show that the two additional interaction variables are not statistically significant. We think that the role of union membership as a factor moderating the effects of industrial automation is an interesting topic for future research.

## 7. Conclusions

The swift pace of introduction of industrial robots, AI and smart machines in production activities in recent years represents a new major process of Schumpeterian creative destruction. This process will in the near future lead to dramatic consequences for employment in many sectors and regions, and it will at the same time create new unprecedented opportunities for productivity growth, wealth and well-being. As for other major transformations in the past, this structural change and the related transition and adjustment process will arguably not be smooth and swift: it will unfold over a period of several years, and it will lead to important negative impacts in the short-run before the long-run economic and societal benefits will eventually emerge.

Studying the effects of automation on employment, extant research has so far mostly focused on aggregate impacts that industrial robots and AI have on employment demand and wages for different industries and countries. The present paper has argued that it is important to shift the focus to the micro-level of analysis and study the impacts of automation technologies on individual workers’ well-being. Specifically, we have put forward the idea that the relevant impacts that it is important to study are not only pecuniary (i.e. related to workers’ employment conditions and wages) but also nonpecuniary (i.e. related to workers’ expectations and future job prospects). *Ceteris paribus*, workers that fear that their working tasks might be replaced by a smart machine in the future may have a lower job satisfaction at present than workers who have more secure job prospects and less uncertainty about the future.

We have investigated this idea by considering a large sample of workers in Norway for the period 2016–2019, and studying the extent to which the introduction of industrial robots in local labor markets affect workers’ fear of being replaced in the future, and in this way hamper their subjective well-being. Our data and results provide a quite striking picture. 40% of Norwegian workers in our sample think their working tasks might be replaced by a machine, and our analysis shows that this fear of replacement significantly lowers their job satisfaction at present. We also find that this transmission mechanism is driven by low-skilled workers, which are those carrying out routine-based tasks, and who are therefore aware to be more exposed to the risks of automation. On the whole, we think that our empirical findings are not only relevant for Norway (the country to which our dataset refers), but they can in principle have more general lessons for other countries too. Automation is by now an important trend that is rapidly diffusing worldwide, and its effect on workers’ health and well-being is therefore a topic of high societal relevance. Schwabe (2019) provides related evidence using a different dataset for a larger sample of European countries. The present work calls therefore for further research that may investigate and extend this research topic in a variety of different countries and continents.

A first important policy implication of our results is that the current process of structural change and creative destruction will in the short-run likely lead to stronger fear of replacement and uncertainty about the future for low-skilled workers carrying out routine work in factories, thus possibly leading to further polarization not only in terms of employment and wages, but also in terms of subjective well-being. To mitigate these negative consequences, which are already visible at present, national authorities should actively support training and re-training policies in such a way that workers that are exposed to future replacement may build up new competencies that can increase their ability to work with smart machines, as well as increase their qualifications and the likelihood to find a new job if this will become necessary in the future. If fear of replacement triggers workers to participate in such training is an interesting question for future studies. In other words, by giving better future prospects to more vulnerable workers, training policies will also contribute to enhance their subjective well-being at present.

Our results also suggest a second reflection and possible policy implication. As noted above, 40% of Norwegian workers in our sample think that their working tasks might be replaced by a machine. According to the Eurobarometer survey, the extent of fear of replacement is roughly the same for workers in other European countries [12]. This number is quite high indeed. Is it reasonable that so many workers fear competition from smart machines, and why is it so?

Extant research on automation and employment has not yet reached a consensus on the direction and size of these effects, and it still presents a vivid debate between those that emphasize negative consequences and those that point out positive economic and societal effects. Hence, there is no clear scientific evidence and consensus at present that could provide the basis for individual workers to form rational and well-informed assessments and expectations about their job prospects in the future. It is therefore reasonable to ask whether the generalized fear of competition from smart machines is actually exaggerated and not based on extant research and established knowledge. The concrete risk is that–in the current phase of rapid and disruptive technological change–societal debates in the media on robots, automation and AI may tend to exaggerate risks and depict gloomy future scenarios, while often neglecting possible long-run benefits for the economy and the society, which are indeed even hard to imagine at the moment [1].

Since media debates on this topic are often biased and tend to overemphasize the negative impacts of automation (which are arguably more “catchy” and attractive for the uninformed audience), this may contribute to explain why so many workers report to fear future machine replacement. However, our paper has shown that such subjective individual assessments about the future may indeed hamper job satisfaction at present. This can also lead to anxiety, mental stress and low motivation at work, which may in turn depress creativity, productivity and innovation in the workplace.

In short, we should not disregard the possibility that a biased and uninformed presentation of this topic in the media may indeed have concrete negative consequences on workers’ subjective well-being by affecting their beliefs about future job prospects. The policy implications of this are certainly not easy to draw. A major point, though, is to stress the importance of having better informed societal debates in the media, and particularly in State-owned channels, that take a more balanced view of the negative and positive consequences of automation, and that avoid spreading fears and gloomy scenarios that are not based on solid evidence and arguments.

## Supporting information

S1 File Online appendix: Additional information and robustness tests. Containing Tables A.1 to A.15.
(DOCX)

## References

[1] Mokyr J., Vickers C. and Ziebarth N.L., The History of Technological Anxiety and the Future of Economic Growth: Is This Time Different? Journal of Economic Perspectives, 2015 29(3): p. 31–50.

[2] Frank M.R., et al, Toward understanding the impact of artificial intelligence on labor. Proceedings of the National Academy of Sciences, 2019 116(14): p. 6531–6539. 10.1073/pnas.1900949116 30910965 PMC6452673

[3] Autor D.H. & Dorn D., The Growth of Low-Skill Service Jobs and the Polarization of the US Labor Market. The American Economic Review, 2013 103(5): p. 1553–1597.

[4] Goos M., Manning A. and Salomons A., Explaining Job Polarization: Routine-Biased Technological Change and Offshoring. American Economic Review, 2014 104(8): p. 2509–26.

[5] Acemoglu, D. & P. Restrepo, Artificial Intelligence, Automation and Work. NBER Working Paper (No. w24196), 2018.

[6] Kaplan G. & Schulhofer-Wohl S., The Changing (Dis-)Utility of Work. Journal of Economic Perspectives, 2018 32(3): p. 239–58. 30362697

[7] Erdogan B., et al, Whistle While You Work: A Review of the Life Satisfaction Literature. Journal of management, 2012 38(4): p. 1038–1083.

[8] Clark A., Oswald A. and Warr P., Is job satisfaction U‐shaped in age? Journal of occupational and organizational psychology, 1996 69(1): p. 57–81.

[9] Böckerman P., Ilmakunnas P. and Johansson E., Job security and employee well-being: Evidence from matched survey and register data. Labour Economics, 2011 18(4): p. 547–554.

[10] Oswald A.J., Proto E. and Sgroi D., Happiness and Productivity. Journal of Labor Economics, 2015 33(4): p. 789–822.

[11] Abeliansky, A.L. & M. Beulmann. (2019). Are they coming for us? Industrial robots and the mental health of workers. cege Discussion Papers, No. 379. University of Göttingen, Center for European, Governance and Economic Development Research (cege), Göttingen.

[12] Schwabe, H., Automation, Fear of Replacement and the Subjective Well-Being of Workers. TIK working paper, 2019.

[13] Rajan K. & Saffiotti A., Towards a science of integrated AI and Robotics. Artificial Intelligence: Special Issue on AI and Robotics, 2017 **247**: p. 1–9.

[14] Acemoglu, D. & D. Autor, Skills, tasks and technologies: Implications for employment and earnings, in Handbook of labor economics. 2011, Elsevier. p. 1043–1171.

[15] Sachs, J.D. & L.J. Kotlikoff. (2012). Smart Machines and Long-Term Misery. NBER Working Paper (No. w18629). National Bureau of Economic Research.

[16] Autor D.H., Why Are There Still So Many Jobs? The History and Future of Workplace Automation. Journal of Economic Perspectives, 2015 29(3): p. 3–30.

[17] Houseman S.N. (2018). Understanding the decline of US manufacturing employment. W.E. Upjohn Institute for Employment Research. Upjohn Institute working paper.

[18] McGuinness, S., K. Pouliakas and P. Redmond, Skills-Displacing Technological Change and Its Impact on Jobs: Challenging Technological Alarmism? IZA Discussion Paper No. 12541, 2019.

[19] Klenert, D., E. Fernandez-Macias and J.-I. Anton. (2020). Do robots really destroy jobs? Evidence from Europe. Seville: European Commission. Joint Research Centre.

[20] Blanas S., Gancia G. and Lee S.Y.T.. (2019). Who is afraid of machines? Barcelone GSE Working Paper Series.

[21] Beaudry P., Green D.A. and Sand B.M., The great reversal in the demand for skill and cognitive tasks. Journal of Labor Economics, 2016 34(S1): p. S199–S247.

[22] Acemoglu D. & Restrepo P., Robots and Jobs: Evidence from US Labor Markets. Journal of Political Economy, 2020 128(6): p. 2188–2244.

[23] Acemoglu, D. & P. Restrepo. (2020). Unpacking Skill Bias: Automation and New Tasks. National Bureau of Economic Research.

[24] Bessen, J., et al. (2020). Automation: A Guide for Policymakers. Retrieved from https://www.brookings.edu/wp-content/uploads/2020/01/Bessen-et-al_Full-report.pdf.

[25] Castellacci F. & Viñas-Bardolet C., Internet use and job satisfaction. Computers in Human Behavior, 2019 90: p. 141–152.

[26] Green F. & Zhu Y., Overqualification, job dissatisfaction and increasing dispersion in the returns to graduate education. Oxford economic papers, 2010 62(4): p. 740–763.

[27] Sousa-Poza A. & Sousa-Poza A.A., Well-being at work: a cross-national analysis of the levels and determinants of job satisfaction. The journal of socio-economics, 2000 29(6): p. 517–538.

[28] Salvatori A., Labour contract regulations and workers' wellbeing: International longitudinal evidence. Labour Economics, 2010 17(4): p. 667–678.

[29] Kahneman D. & Tversky A., Prospect Theory: An Analysis of Decision under Risk. Econometrica, 1979 47(2): p. 263–291.

[30] Belfield C.R. & Harris R.D., How well do theories of job matching explain variations in job satisfaction across education levels? Evidence for UK graduates. Applied economics, 2002 34(5): p. 535–548.

[31] Aghion P., et al, Creative destruction and subjective well-being. American Economic Review, 2016 106(12): p. 3869–97. 10.1257/aer.20150338 28713168 PMC5510036

[32] IFR. (2017). World Robotics: Industrial Robots. International Federation of Robotics.

[33] IFR. (2018). World Robotics 2018: Industrial Robots. Retrieved from International Federation of Robotics: https://ifr.org/.

[34] Chiacchio, F., G. Petropoulos and D. Pichler, The impact of industrial robots on EU employment and wages: A local labour market approach. Bruegel Working Papers (02), 2018.

[35] Graetz G. & Michaels G., Robots at work. The Review of Economics and Statistics, 2018 100(5): p. 753–768.

[36] Dauth, W., et al., Adjusting to Robots: Worker-Level Evidence. Opportunity and Inclusive Growth Institute Working Paper (13), 2018.

[37] Michaels G., Natraj A. and Van Reenen J., Has ICT Polarized Skill Demand? Evidence from Eleven Countries over Twenty-Five Years. Review of Economics and Statistics, 2014 96(1): p. 60–77.

[38] Falk M. & Biagi F., Relative demand for highly skilled workers and use of different ICT technologies. Applied Economics, 2017 49(9): p. 903–914.

[39] Bhuller M., et al, Broadband internet: An information superhighway to sex crime? Review of Economic Studies, 2013 80(4): p. 1237–1266.

[40] Akerman A., Gaarder I. and Mogstad M., The skill complementarity of broadband internet. The Quarterly Journal of Economics, 2015 130(4): p. 1781–1824.

[41] Angrist J.D. & Pischke J.-S., Mostly harmless econometrics: An empiricist's companion. Vol. 2009, Princeton, NJ.: Princeton university press.

[42] European Commission. Digital Single Market. 2014; Available from: https://europa.eu/european-union/file/1497/.

[43] Frank M.R., et al, Small cities face greater impact from automation. Journal of the Royal Society Interface, 2018 15(139): p. 20170946 10.1098/rsif.2017.0946 29436514 PMC5832739

[44] Acemoglu, D. & P. Restrepo, Demographics and Automation. NBER Working Paper (No. w24421), 2018.

[45] Card D., et al, Inequality at work: The effect of peer salaries on job satisfaction. American Economic Review, 2012 102(6): p. 2981–3003.

[46] Sajaia Z., Maximum likelihood estimation of a bivariate ordered probit model: implementation and Monte Carlo simulations. The Stata Journal, 2008 4(2): p. 1–18.

[47] Monfardini C. & Radice R., Testing exogeneity in the bivariate probit model: A Monte Carlo study. Oxford Bulletin of Economics and Statistics, 2008 70(2): p. 271–282.

[48] Wooldridge, J.M., Econometric analysis of cross section and panel data. 2010: MIT press.

[49] Maddala, G.S. & L.-F. Lee, Recursive models with qualitative endogenous variables, in Annals of Economic and Social Measurement, Volume 5, number 4. 1976, NBER. p. 525–545.

[50] Roodman D., Fitting fully observed recursive mixed-process models with cmp. Stata Journal, 2011 11(2): p. 159–206.

[51] Moulton B.R., Random group effects and the precision of regression estimates. Journal of Econometrics, 1986 32(3): p. 385–397.

[52] Cameron C.A. & Miller D.L., A practitioner’s guide to cluster-robust inference. Journal of Human Resources, 2015 50(2): p. 317–372.

[53] Becker T.E., Potential Problems in the Statistical Control of Variables in Organizational Research: A Qualitative Analysis With Recommendations. Organizational Research Methods, 2005 8(3): p. 274–289.

[54] Spector P.E. & Brannick M.T., Methodological Urban Legends: The Misuse of Statistical Control Variables. Organizational Research Methods, 2011 14(2): p. 287–305.

[55] Hunter J.E., Schmidt F.L. and Judiesch M.K., Individual differences in output variability as a function of job complexity. Journal of Applied Psychology, 1990 75(1): p. 28–42.

[56] Acemoglu, D., P. Aghion and G.L. Violante. Deunionization, technical change and inequality. in Carnegie-Rochester conference series on public policy. 2001. Elsevier.

