# Supplementary material for: Automation, workers’ skills and job satisfaction
Source: PLoS One. 2020 Nov 30;15(11):e0242929. doi: 10.1371/journal.pone.0242929 (PMC7703879; doi:10.1371/journal.pone.0242929)
Supplement: S1 File — Containing Tables A.1 to A.15. (DOCX) [file pone.0242929.s001.docx]

**Supporting information file**

**Automation, workers’ skills and job satisfaction**

**Henrik Schwabe and Fulvio Castellacci^*^**

University of Oslo,

Postboks 1108, 0317 Oslo, Norway

^*^Corresponding author: fulvio.castellacci@tik.uio.no

Manuscript revised and resubmitted to *PLOS ONE*, 13 October 2020

**Online appendix:**

**Additional information and robustness tests**

**Table A1: Firm adoption of industrial and service robots in Norwegian firms.**

| **Use industrial robots** |  |
| --- | --- |
| All enterprises | 3 % |
| SME | 3 % |
| Large | 16 % |
|  |  |
| **Use service robots** |  |
| All enterprises | 1 % |
| SME | 1 % |
| Large | 10 % |
|  |  |
| **Use industrial or service robots** |  |
| All enterprises | 4 % |
| SME | 3 % |
| Large | 23 % |

All enterprises, without financial sector: 10 persons employed or more. SMEs, without financial sector: 10-249 persons employed. Large enterprises, without financial: 250 persons employed or more. Data source: Eurostat, ICT (Information and Communication Technologies) usage and e-commerce in enterprises 2018.

**Table A2: Purpose of use for industrial robots (operational stock).**

|  | **1999** | **2017** |
| --- | --- | --- |
| 000 - All Applications | 100 % | 100 % |
| 110 - Handling operations/Machine Tending | 36% | 59% |
| 160 - Welding and soldering | 27% | 12% |
| 170 - Dispensing | 6% | 1% |
| 190 - Processing | 19 % | 3% |
| 200 - Assembling and disassembling | 4% | 2% |
| 900 - Other | 3% | 5% |

Data source: The International Federation of Robotics (IFR).

**Table A3: Purpose of use for service robots (operational stock).**

|  |  |  |  |
| --- | --- | --- | --- |
|  | **All enterprises** | **SME** | **Large** |
| Surveillance, security or inspection tasks | 22 % | 23 % | 20 % |
| Transportation of people or goods | 17 % | 16 % | 24 % |
| Cleaning or waste disposal tasks | 19 % | 20 % | 18 % |
| Warehouse management systems | 22 % | 19 % | 39 % |
| Assembly works | 12 % | 14 % | 4 % |
| Robotic store clerk tasks | 20 % | 17 % | 31 % |
| Construction works or damage repair tasks | 14 % | 17 % | 2 % |
| Any of the listed purposes | 84 % | 82 % | 93 % |

Data source: Eurostat, ICT (Information and Communication Technologies) usage and e-commerce in enterprises 2016.

**Table A4: The use, source and employment of Big Data analysis in Norwegian Firms.**

|  | Share of firms |  | Big Data source |  |  |  |  | Analysts |  |
| --- | --- | --- | --- | --- | --- | --- | --- | --- | --- |
|  | Analyzing Big Data |  | Smart devices/ sensors | Geo-data of portable devices | Social Media | Other sources |  | Internal | External |
| All enterprises | 15 % |  | 33 % | 33 % | 62 % | 28 % |  | 59 % | 36 % |
| SMEs | 15 % |  | 32 % | 33 % | 63 % | 27 % |  | 57 % | 36 % |
| Large | 39 % |  | 55 % | 43 % | 43 % | 52 % |  | 82 % | 42 % |
|  |  |  |  |  |  |  |  |  |  |

Percentage of all enterprises, without financial sector (10 persons employed or more). Data source: Eurostat, ICT (Information and Communication Technologies) usage and e-commerce in enterprises 2016.

**Table A5: First and second stage results. Machine replacement, annual levels.**

|  | (1) | (2) | (3) | (4) |
| --- | --- | --- | --- | --- |
|  | OLS | Probit | 2SLS | IVOPROBIT |
|  | 1^st^ stage | 1^st^ stage | 2^nd^ stage | 2^nd^ stage |
| Robot adoption | 0.091** | 0.237** |  |  |
|  | (0.042) | (0.108) |  |  |
| Machine replacement |  |  | -2.239** | -1.094*** |
|  |  |  | (1.060) | (0.115) |
| Age | -0.004*** | -0.011*** | 0.001 | 0.007*** |
|  | (0.001) | (0.002) | (0.005) | (0.001) |
| Union membership | -0.054*** | -0.144*** | -0.118* | -0.062*** |
|  | (0.013) | (0.035) | (0.064) | (0.018) |
| Income scale = 2 | -0.092*** | -0.263*** | -0.276*** | -0.145*** |
|  | (0.023) | (0.061) | (0.106) | (0.052) |
| Income scale = 3 | -0.005 | -0.015 | -0.003 | 0.015 |
|  | (0.020) | (0.053) | (0.043) | (0.037) |
| Income scale = 4 | 0.023 | 0.061 | 0.124 | 0.120** |
|  | (0.022) | (0.058) | (0.078) | (0.054) |
| Income scale = 5 | 0.025 | 0.066 | 0.180** | 0.185*** |
|  | (0.030) | (0.078) | (0.084) | (0.051) |
| Income scale = 6 | 0.039 | 0.102 | 0.257** | 0.245*** |
|  | (0.035) | (0.092) | (0.100) | (0.066) |
| Income scale = 7 | 0.074** | 0.195*** | 0.333*** | 0.288*** |
|  | (0.027) | (0.072) | (0.118) | (0.051) |
| Income scale = 8 | 0.035 | 0.092 | 0.267*** | 0.272*** |
|  | (0.028) | (0.074) | (0.098) | (0.073) |
| Income scale = 9 | 0.061 | 0.162 | 0.437*** | 0.433*** |
|  | (0.057) | (0.149) | (0.161) | (0.096) |
| University degree | 0.041* | 0.107* | 0.090 | 0.038 |
|  | (0.022) | (0.058) | (0.056) | (0.029) |
| Woman | -0.009 | -0.023 | 0.077** | 0.125*** |
|  | (0.018) | (0.047) | (0.036) | (0.026) |
| Industry employment | -0.057* | -0.150** | -0.189*** | -0.209*** |
|  | (0.027) | (0.070) | (0.044) | (0.028) |
|  |  |  |  |  |
| Controls |  |  |  |  |
| Regional dummies | ✓ | ✓ | ✓ | ✓ |
| Year dummies | ✓ | ✓ | ✓ | ✓ |
|  |  |  |  |  |
| F-stat | 8.55 |  |  |  |
|  |  |  |  |  |
| N | 10,051 | 10,051 | 10,051 | 10,051 |

Robust standard errors in parentheses are clustered for workers in the same region and industry. Columns 1 and 3 present OLS estimates. Columns 2 and 4 show probit estimates. * p<0.10, ** p<0.05, ***p<0.01

**Table A6: First stage results, controlling for fear of job loss. Dependent variable: machine replacement.**

|  | (1) | (2) | (3) | (4) |
| --- | --- | --- | --- | --- |
|  | OLS | OLS | Probit | Probit |
|  |  |  |  |  |
| Robot adoption | 0.819*** | 0.782*** | 2.168*** | 2.068*** |
|  | (0.257) | (0.263) | (0.689) | (0.700) |
| Job loss worry | 0.074*** |  | 0.198*** |  |
|  | (0.013) |  | (0.036) |  |
| Unemployed in 5 years |  | 0.154*** |  | 0.402*** |
|  |  | (0.032) |  | (0.082) |
| Age | -0.004*** | -0.004*** | -0.010*** | -0.011*** |
|  | (0.001) | (0.001) | (0.002) | (0.002) |
| Union membership | -0.057*** | -0.058*** | -0.151*** | -0.154*** |
|  | (0.013) | (0.013) | (0.036) | (0.034) |
| Income scale = 2 | -0.095*** | -0.104*** | -0.272*** | -0.296*** |
|  | (0.023) | (0.026) | (0.062) | (0.073) |
| Income scale = 3 | -0.002 | -0.010 | -0.007 | -0.029 |
|  | (0.017) | (0.016) | (0.044) | (0.041) |
| Income scale = 4 | 0.026 | 0.019 | 0.069 | 0.049 |
|  | (0.019) | (0.015) | (0.052) | (0.039) |
| Income scale = 5 | 0.031 | 0.026 | 0.084 | 0.067 |
|  | (0.025) | (0.019) | (0.067) | (0.051) |
| Income scale = 6 | 0.045 | 0.036 | 0.119 | 0.092 |
|  | (0.033) | (0.025) | (0.087) | (0.067) |
| Income scale = 7 | 0.081*** | 0.082*** | 0.215*** | 0.212*** |
|  | (0.023) | (0.019) | (0.062) | (0.050) |
| Income scale = 8 | 0.041 | 0.035 | 0.107 | 0.091 |
|  | (0.026) | (0.028) | (0.069) | (0.074) |
| Income scale = 9 | 0.068 | 0.065 | 0.179 | 0.168 |
|  | (0.051) | (0.045) | (0.133) | (0.117) |
| University degree | 0.046* | 0.040* | 0.124** | 0.107** |
|  | (0.022) | (0.020) | (0.059) | (0.053) |
| Woman | -0.008 | -0.010 | -0.019 | -0.026 |
|  | (0.015) | (0.018) | (0.041) | (0.047) |
| Industry employment | -0.044*** | -0.052*** | -0.120*** | -0.140*** |
|  | (0.015) | (0.016) | (0.040) | (0.043) |
|  |  |  |  |  |
| Controls |  |  |  |  |
| Regional dummies | ✓ | ✓ | ✓ | ✓ |
| Year dummies | ✓ | ✓ | ✓ | ✓ |
|  |  |  |  |  |
| N | 9,829 | 9,184 | 9,829 | 9,184 |

Robust standard errors in parentheses are clustered for workers in the same region and industry. Columns 1 and 2 present OLS estimates. Columns 3 and 4 show probit estimates. * p<0.10, ** p<0.05, *** p<0.01

**Table A7: Second stage results, controlling for fear of job loss. Dependent variable: Job satisfaction.**

|  | (1) | (2) | (3) | (4) |
| --- | --- | --- | --- | --- |
|  | 2SLS | IVOPROBIT | 2SLS | IVOPROBIT |
| Machine replacement | -0.567** | -1.050*** | -0.775** | -1.048*** |
|  | (0.250) | (0.142) | (0.337) | (0.144) |
| Job loss worry | -0.299*** | -0.340*** |  |  |
|  | (0.029) | (0.034) |  |  |
| Unemployed in 5 years |  |  | -0.365*** | -0.355*** |
|  |  |  | (0.066) | (0.065) |
| Age | 0.006*** | 0.006*** | 0.006*** | 0.008*** |
|  | (0.001) | (0.002) | (0.002) | (0.002) |
| Union membership | -0.037* | -0.068*** | -0.039 | -0.058*** |
|  | (0.022) | (0.019) | (0.024) | (0.019) |
| Income scale = 2 | -0.089 | -0.117** | -0.146* | -0.156** |
|  | (0.065) | (0.053) | (0.075) | (0.067) |
| Income scale = 3 | 0.023 | 0.032 | 0.008 | 0.012 |
|  | (0.031) | (0.031) | (0.050) | (0.052) |
| Income scale = 4 | 0.085 | 0.115** | 0.105* | 0.132** |
|  | (0.052) | (0.057) | (0.061) | (0.064) |
| Income scale = 5 | 0.130*** | 0.176*** | 0.140*** | 0.178*** |
|  | (0.046) | (0.053) | (0.041) | (0.042) |
| Income scale = 6 | 0.181*** | 0.240*** | 0.176*** | 0.219*** |
|  | (0.061) | (0.072) | (0.062) | (0.069) |
| Income scale = 7 | 0.196*** | 0.288*** | 0.210*** | 0.280*** |
|  | (0.055) | (0.057) | (0.060) | (0.058) |
| Income scale = 8 | 0.193*** | 0.273*** | 0.202*** | 0.269*** |
|  | (0.061) | (0.072) | (0.061) | (0.069) |
| Income scale = 9 | 0.330*** | 0.463*** | 0.333*** | 0.443*** |
|  | (0.071) | (0.097) | (0.074) | (0.099) |
| University degree | -0.002 | 0.002 | 0.028 | 0.027 |
|  | (0.020) | (0.027) | (0.025) | (0.029) |
| Woman | 0.080*** | 0.114*** | 0.081*** | 0.114*** |
|  | (0.018) | (0.027) | (0.020) | (0.027) |
| Industry employment | -0.143*** | -0.186*** | -0.192*** | -0.237*** |
|  | (0.018) | (0.024) | (0.023) | (0.028) |
|  |  |  |  |  |
| Controls |  |  |  |  |
| Regional dummies | ✓ | ✓ | ✓ | ✓ |
| Year dummies | ✓ | ✓ | ✓ | ✓ |
|  |  |  |  |  |
| N | 9,829 | 9,829 | 9,184 | 9,184 |

Robust standard errors in parentheses are clustered for workers in the same region and industry. * p<0.10, ** p<0.05, *** p<0.01

**Table A8: First stage results, controlling for “employability”. Dependent variable: machine replacement.**

|  | (2) | (3) | (5) | (6) |
| --- | --- | --- | --- | --- |
|  | OLS | OLS | PROBIT | PROBIT |
|  |  |  |  |  |
| Robot adoption | 0.825*** | 0.910*** | 2.174*** | 2.408*** |
|  | (0.262) | (0.282) | (0.697) | (0.756) |
| Difficult to find new job | 0.031* |  | 0.085* |  |
|  | (0.017) |  | (0.045) |  |
| Insufficient skills in current job |  | 0.050*** |  | 0.131*** |
|  |  | (0.010) |  | (0.026) |
| Age | -0.004*** | -0.004*** | -0.011*** | -0.010*** |
|  | (0.001) | (0.001) | (0.002) | (0.002) |
| Union membership | -0.060*** | -0.058*** | -0.160*** | -0.155*** |
|  | (0.013) | (0.014) | (0.035) | (0.038) |
| Income scale = 2 | -0.098*** | -0.094*** | -0.279*** | -0.269*** |
|  | (0.032) | (0.026) | (0.084) | (0.070) |
| Income scale = 3 | -0.011 | -0.015 | -0.031 | -0.042 |
|  | (0.026) | (0.021) | (0.068) | (0.056) |
| Income scale = 4 | 0.017 | 0.018 | 0.045 | 0.046 |
|  | (0.023) | (0.024) | (0.062) | (0.063) |
| Income scale = 5 | 0.021 | 0.020 | 0.057 | 0.053 |
|  | (0.027) | (0.031) | (0.071) | (0.082) |
| Income scale = 6 | 0.035 | 0.037 | 0.093 | 0.098 |
|  | (0.035) | (0.038) | (0.091) | (0.100) |
| Income scale = 7 | 0.078** | 0.071** | 0.204*** | 0.188** |
|  | (0.029) | (0.028) | (0.076) | (0.073) |
| Income scale = 8 | 0.034 | 0.029 | 0.089 | 0.073 |
|  | (0.026) | (0.030) | (0.071) | (0.079) |
| Income scale = 9 | 0.069 | 0.061 | 0.181 | 0.159 |
|  | (0.054) | (0.055) | (0.139) | (0.143) |
| University degree | 0.041* | 0.034 | 0.109* | 0.091 |
|  | (0.022) | (0.021) | (0.058) | (0.056) |
| Woman | -0.012 | -0.012 | -0.030 | -0.030 |
|  | (0.018) | (0.017) | (0.047) | (0.046) |
| Industry employment | -0.047** | -0.050*** | -0.126*** | -0.134*** |
|  | (0.018) | (0.016) | (0.048) | (0.043) |
|  |  |  |  |  |
| Controls |  |  |  |  |
| Regional dummies | ✓ | ✓ | ✓ | ✓ |
| Year dummies | ✓ | ✓ | ✓ | ✓ |
|  |  |  |  |  |
| F-stat | 9.94 | 10.4 |  |  |
|  |  |  |  |  |
| N | 9,451 | 9,890 | 9,451 | 9,890 |

Robust standard errors in parentheses are clustered for workers in the same region and industry. * p<0.10, ** p<0.05, ***p<0.01

**Table A9: First stage results, controlling for “employability”. Regressions by education level. Dependent variable: machine replacement.**

|  | (1) | (2) | (3) | (4) |
| --- | --- | --- | --- | --- |
|  | No university education | University education | No university education | University education |
| Robot adoption | 1.032*** | 0.623* | 1.097*** | 0.811*** |
|  | (0.318) | (0.304) | (0.332) | (0.254) |
| Difficult to find new job | 0.042** | 0.017 |  |  |
|  | (0.019) | (0.019) |  |  |
| Insufficient skills in current job |  |  | 0.042** | 0.058*** |
|  |  |  | (0.014) | (0.012) |
| Age | -0.004*** | -0.004*** | -0.004*** | -0.004*** |
|  | (0.001) | (0.001) | (0.001) | (0.001) |
| Union membership | -0.027 | -0.089*** | -0.025 | -0.087*** |
|  | (0.021) | (0.020) | (0.020) | (0.021) |
| Income scale = 2 | -0.122** | -0.032 | -0.120** | -0.035 |
|  | (0.050) | (0.044) | (0.042) | (0.026) |
| Income scale = 3 | 0.001 | -0.028 | -0.008 | -0.030 |
|  | (0.058) | (0.043) | (0.049) | (0.032) |
| Income scale = 4 | 0.028 | 0.024 | 0.029 | 0.026 |
|  | (0.052) | (0.023) | (0.047) | (0.019) |
| Income scale = 5 | 0.085 | 0.002 | 0.083 | 0.005 |
|  | (0.072) | (0.024) | (0.068) | (0.027) |
| Income scale = 6 | 0.020 | 0.054 | 0.013 | 0.066* |
|  | (0.049) | (0.036) | (0.048) | (0.037) |
| Income scale = 7 | 0.108 | 0.071* | 0.091 | 0.075** |
|  | (0.072) | (0.039) | (0.063) | (0.034) |
| Income scale = 8 | 0.071 | 0.016 | 0.048 | 0.024 |
|  | (0.057) | (0.038) | (0.051) | (0.036) |
| Income scale = 9 | 0.065 | 0.065 | 0.061 | 0.060 |
|  | (0.063) | (0.059) | (0.057) | (0.063) |
| Woman | 0.049* | -0.055*** | 0.059** | -0.063*** |
|  | (0.023) | (0.018) | (0.023) | (0.019) |
| Industry employment | -0.066*** | -0.001 | -0.065*** | -0.005 |
|  | (0.021) | (0.033) | (0.017) | (0.033) |
|  |  |  |  |  |
| Controls |  |  |  |  |
| Regional dummies | ✓ | ✓ | ✓ | ✓ |
| Year dummies | ✓ | ✓ | ✓ | ✓ |
|  |  |  |  |  |
| N | 4,117 | 5,334 | 4,335 | 5,555 |
|  |  |  |  |  |

Robust standard errors in parentheses are clustered for workers in the same region and industry. The table present results from OLS estimations. * p<0.10, ** p<0.05, *** p<0.01

**Table A10: Marginal effects of machine replacement on job satisfaction for workers of different education levels and of different ages.**

|  |  |  |  |  |
| --- | --- | --- | --- | --- |
|  | Controlling for:  Difficult to find new job |  | Controlling for:  Insufficient skill in current job |  |
|  | Below university education | University education | Below university education | University education |
|  |  |  |  |  |
| Age group  <30 years | 0.107 | 1.666 | 0.211 | 1.145 |
|  | (0.355) | (2.216) | (0.408) | (2.874) |
| 30-44 years | -0.495* | -0.687 | -0.398*** | -1.229** |
|  | (0.279) | (6.235) | (0.147) | (0.484) |
| 45-59 years | -0.322* | 0.192 | -0.359** | 0.108 |
|  | (0.170) | (0.449) | (0.181) | (0.432) |
| 60+ years | -0.949*** | -0.622 | -1.069*** | -0.581 |
|  | (0.252) | (1.721) | (0.327) | (1.179) |
|  |  |  |  |  |
| Controls |  |  |  |  |
| Individual controls | ✓ | ✓ | ✓ | ✓ |
| Regional dummies | ✓ | ✓ | ✓ | ✓ |
| Year dummies | ✓ | ✓ | ✓ | ✓ |
|  |  |  |  |  |
| N | 9,451 | 9,451 | 9,890 | 9,890 |

Robust standard errors in parentheses are clustered for workers in the same region and industry. The table present results from bivariate recursive probit estimations. * p<0.10, ** p<0.05, *** p<0.01

**Table A11: First stage results. Robustness tests.**

|  | (1) | (2) | (3) | (4) | (5) | (6) |
| --- | --- | --- | --- | --- | --- | --- |
|  | Machine replacement | Machine replacement | Machine replacement | Machine replacement | Machine replacement | Machine replacement |
| Robot exposure | 0.830*** | 0.831*** | 0.858*** | 0.860*** | 0.861*** | 0.860*** |
|  | (0.262) | (0.265) | (0.260) | (0.261) | (0.263) | (0.264) |
| Age | -0.004*** | -0.004*** | -0.004*** | -0.004*** | -0.004*** | -0.004*** |
|  | (0.001) | (0.001) | (0.001) | (0.001) | (0.001) | (0.001) |
| Union membership | -0.056*** | -0.056*** | -0.056*** | -0.056*** | -0.056*** | -0.056*** |
|  | (0.013) | (0.013) | (0.013) | (0.013) | (0.013) | (0.013) |
| Income scale = 2 | -0.094*** | -0.094*** | -0.094*** | -0.094*** | -0.094*** | -0.094*** |
|  | (0.024) | (0.024) | (0.025) | (0.025) | (0.025) | (0.025) |
| Income scale = 3 | -0.007 | -0.007 | -0.008 | -0.008 | -0.008 | -0.008 |
|  | (0.021) | (0.021) | (0.021) | (0.021) | (0.021) | (0.021) |
| Income scale = 4 | 0.023 | 0.023 | 0.023 | 0.023 | 0.023 | 0.023 |
|  | (0.022) | (0.022) | (0.022) | (0.022) | (0.022) | (0.022) |
| Income scale = 5 | 0.027 | 0.027 | 0.027 | 0.027 | 0.027 | 0.027 |
|  | (0.029) | (0.029) | (0.029) | (0.029) | (0.029) | (0.029) |
| Income scale = 6 | 0.042 | 0.042 | 0.042 | 0.042 | 0.042 | 0.042 |
|  | (0.034) | (0.034) | (0.033) | (0.033) | (0.033) | (0.034) |
| Income scale = 7 | 0.078*** | 0.078*** | 0.078*** | 0.078*** | 0.078*** | 0.078*** |
|  | (0.025) | (0.026) | (0.026) | (0.026) | (0.026) | (0.026) |
| Income scale = 8 | 0.038 | 0.038 | 0.038 | 0.038 | 0.038 | 0.038 |
|  | (0.026) | (0.026) | (0.025) | (0.025) | (0.025) | (0.025) |
| Income scale = 9 | 0.069 | 0.069 | 0.070 | 0.069 | 0.069 | 0.069 |
|  | (0.052) | (0.052) | (0.053) | (0.053) | (0.053) | (0.052) |
| University degree | 0.037* | 0.037* | 0.037* | 0.037* | 0.037* | 0.037* |
|  | (0.021) | (0.021) | (0.021) | (0.021) | (0.021) | (0.021) |
| Woman | -0.011 | -0.011 | -0.011 | -0.011 | -0.011 | -0.011 |
|  | (0.017) | (0.017) | (0.017) | (0.017) | (0.017) | (0.017) |
| Industry employment | -0.044** | -0.044** | -0.044** | -0.044** | -0.045** | -0.044** |
|  | (0.016) | (0.016) | (0.016) | (0.016) | (0.016) | (0.016) |
| GDP | -0.304 | -0.279 | -0.252 | -0.357 | -0.412 | 0.139 |
|  | (0.353) | (0.354) | (0.315) | (0.434) | (0.659) | (1.273) |
| % pop. with tertiary educ. |  | 0.004 | 0.007 | 0.006 | 0.005 | 0.010 |
|  |  | (0.014) | (0.013) | (0.012) | (0.011) | (0.011) |
| Broadband availability in business properties |  |  | -0.397*** | -0.405*** | -0.405*** | -0.069 |
|  |  |  | (0.116) | (0.121) | (0.121) | (0.442) |
| Unemployment benefit recipients |  |  |  | -0.017 | -0.025 | 0.091 |
|  |  |  |  | (0.040) | (0.064) | (0.195) |
| % large industrial firms |  |  |  |  | -0.293 | 1.910 |
|  |  |  |  |  | (1.419) | (4.207) |
| Log(population) in region |  |  |  |  |  | 3.069 |
|  |  |  |  |  |  | (4.145) |
|  |  |  |  |  |  |  |
| F-stat | 10.05 | 9.89 | 10.92 | 10.85 | 10.75 | 10.66 |
|  |  |  |  |  |  |  |
| N | 10,051 | 10,051 | 10,051 | 10,051 | 10,051 | 10,051 |

Robust standard errors in parentheses are clustered for workers in the same region and industry. The table present results from OLS estimations. * p<0.10, ** p<0.05, *** p<0.01

**Table A12: Second stage results. Robustness tests.**

|  | (1) | (2) | 3) | (4) | (5) | (6) |
| --- | --- | --- | --- | --- | --- | --- |
|  | Job satisfaction | Job satisfaction | Job satisfaction | Job satisfaction | Job satisfaction | Job satisfaction |
| Machine replacement | -0.993*** | -0.993*** | -1.000*** | -1.006*** | -1.004*** | -1.000*** |
|  | (0.174) | (0.176) | (0.171) | (0.165) | (0.165) | (0.165) |
| Age | 0.008*** | 0.008*** | 0.008*** | 0.008*** | 0.008*** | 0.008*** |
|  | (0.002) | (0.002) | (0.002) | (0.002) | (0.002) | (0.002) |
| Union membership | -0.057*** | -0.057*** | -0.057*** | -0.057*** | -0.057*** | -0.057*** |
|  | (0.021) | (0.021) | (0.020) | (0.020) | (0.020) | (0.020) |
| Income scale = 2 | -0.136*** | -0.135*** | -0.136*** | -0.135** | -0.134** | -0.134** |
|  | (0.052) | (0.052) | (0.053) | (0.053) | (0.053) | (0.054) |
| Income scale = 3 | 0.017 | 0.017 | 0.017 | 0.018 | 0.018 | 0.018 |
|  | (0.038) | (0.038) | (0.038) | (0.039) | (0.039) | (0.039) |
| Income scale = 4 | 0.121** | 0.120** | 0.121** | 0.121** | 0.121** | 0.121** |
|  | (0.055) | (0.055) | (0.055) | (0.055) | (0.055) | (0.055) |
| Income scale = 5 | 0.188*** | 0.188*** | 0.188*** | 0.188*** | 0.188*** | 0.188*** |
|  | (0.049) | (0.049) | (0.050) | (0.050) | (0.049) | (0.049) |
| Income scale = 6 | 0.247*** | 0.247*** | 0.247*** | 0.248*** | 0.248*** | 0.248*** |
|  | (0.067) | (0.067) | (0.067) | (0.067) | (0.067) | (0.067) |
| Income scale = 7 | 0.286*** | 0.286*** | 0.286*** | 0.287*** | 0.287*** | 0.287*** |
|  | (0.054) | (0.054) | (0.054) | (0.054) | (0.054) | (0.054) |
| Income scale = 8 | 0.275*** | 0.275*** | 0.275*** | 0.275*** | 0.275*** | 0.275*** |
|  | (0.074) | (0.074) | (0.074) | (0.074) | (0.074) | (0.074) |
| Income scale = 9 | 0.435*** | 0.435*** | 0.435*** | 0.435*** | 0.434*** | 0.434*** |
|  | (0.095) | (0.095) | (0.095) | (0.095) | (0.095) | (0.095) |
| University degree | 0.034 | 0.034 | 0.034 | 0.034 | 0.035 | 0.034 |
|  | (0.028) | (0.028) | (0.028) | (0.028) | (0.028) | (0.028) |
| Woman | 0.129*** | 0.129*** | 0.129*** | 0.129*** | 0.128*** | 0.129*** |
|  | (0.028) | (0.028) | (0.028) | (0.028) | (0.028) | (0.028) |
| Industry employment | -0.212*** | -0.212*** | -0.212*** | -0.211*** | -0.211*** | -0.211*** |
|  | (0.026) | (0.026) | (0.026) | (0.026) | (0.026) | (0.026) |
| GDP | 0.635 | 0.786 | 0.773 | 1.737** | 1.088** | 0.698 |
|  | (0.502) | (0.596) | (0.579) | (0.699) | (0.536) | (0.691) |
| % pop. with tertiary educ. |  | 0.022 | 0.019 | 0.034 | 0.020 | 0.016 |
|  |  | (0.032) | (0.032) | (0.030) | (0.029) | (0.031) |
| Broadband availability in business properties |  |  | 0.277 | 0.348** | 0.344** | 0.131 |
|  |  |  | (0.202) | (0.167) | (0.168) | (0.175) |
| Unemployment benefit recipients |  |  |  | 0.157 | 0.064 | -0.015 |
|  |  |  |  | (0.132) | (0.137) | (0.135) |
| % large industrial firms |  |  |  |  | -3.529 | -5.024* |
|  |  |  |  |  | (2.813) | (2.667) |
| Log(population) in region |  |  |  |  |  | -1.954** |
|  |  |  |  |  |  | (0.778) |
|  |  |  |  |  |  |  |
| N | 10,051 | 10,051 | 10,051 | 10,051 | 10,051 | 10,051 |

Robust standard errors in parentheses are clustered for workers in the same region and industry. The table present results from bivariate recursive probit estimations. * p<0.10, ** p<0.05, *** p<0.01

**Table A13: Placebo tests (first stage).**

|  | (1) | (2) | (3) | (4) | (5) | (6) | (7) |
| --- | --- | --- | --- | --- | --- | --- | --- |
| Robot exposure | 0.814** | 0.767* | 0.769* | 0.826** | 0.829** | 0.830** | 0.830** |
|  | (0.363) | (0.370) | (0.375) | (0.364) | (0.364) | (0.367) | (0.368) |
| Robot exposure in *t+1* | 0.011 | 0.018 | 0.017 | 0.009 | 0.009 | 0.008 | 0.009 |
|  | (0.066) | (0.066) | (0.066) | (0.065) | (0.065) | (0.065) | (0.065) |
| Age | -0.004*** | -0.004*** | -0.004*** | -0.004*** | -0.004*** | -0.004*** | -0.004*** |
|  | (0.001) | (0.001) | (0.001) | (0.001) | (0.001) | (0.001) | (0.001) |
| Union membership | -0.056*** | -0.056*** | -0.056*** | -0.056*** | -0.056*** | -0.056*** | -0.056*** |
|  | (0.013) | (0.013) | (0.013) | (0.013) | (0.013) | (0.013) | (0.013) |
| Income scale = 2 | -0.093*** | -0.093*** | -0.093*** | -0.094*** | -0.094*** | -0.094*** | -0.094*** |
|  | (0.023) | (0.023) | (0.023) | (0.024) | (0.024) | (0.024) | (0.024) |
| Income scale = 3 | -0.006 | -0.006 | -0.006 | -0.007 | -0.007 | -0.007 | -0.007 |
|  | (0.021) | (0.020) | (0.020) | (0.021) | (0.021) | (0.021) | (0.021) |
| Income scale = 4 | 0.024 | 0.024 | 0.024 | 0.023 | 0.023 | 0.023 | 0.023 |
|  | (0.022) | (0.022) | (0.022) | (0.022) | (0.022) | (0.022) | (0.022) |
| Income scale = 5 | 0.027 | 0.027 | 0.027 | 0.027 | 0.027 | 0.027 | 0.027 |
|  | (0.029) | (0.029) | (0.029) | (0.029) | (0.029) | (0.029) | (0.029) |
| Income scale = 6 | 0.042 | 0.042 | 0.042 | 0.042 | 0.041 | 0.041 | 0.041 |
|  | (0.035) | (0.034) | (0.034) | (0.034) | (0.034) | (0.034) | (0.034) |
| Income scale = 7 | 0.078** | 0.077** | 0.077** | 0.078** | 0.078** | 0.078** | 0.077** |
|  | (0.027) | (0.027) | (0.027) | (0.027) | (0.027) | (0.027) | (0.027) |
| Income scale = 8 | 0.038 | 0.037 | 0.037 | 0.038 | 0.037 | 0.037 | 0.037 |
|  | (0.027) | (0.027) | (0.027) | (0.027) | (0.027) | (0.027) | (0.027) |
| Income scale = 9 | 0.068 | 0.067 | 0.067 | 0.069 | 0.069 | 0.069 | 0.069 |
|  | (0.057) | (0.057) | (0.057) | (0.057) | (0.057) | (0.057) | (0.057) |
| University degree | 0.037* | 0.037* | 0.037* | 0.037* | 0.037 | 0.037* | 0.037* |
|  | (0.021) | (0.021) | (0.021) | (0.021) | (0.021) | (0.021) | (0.021) |
| Woman | -0.011 | -0.011 | -0.011 | -0.011 | -0.011 | -0.011 | -0.011 |
|  | (0.017) | (0.017) | (0.017) | (0.017) | (0.017) | (0.017) | (0.017) |
| Industry employment | -0.047** | -0.048** | -0.048** | -0.047** | -0.047** | -0.047** | -0.047** |
|  | (0.021) | (0.021) | (0.021) | (0.021) | (0.021) | (0.021) | (0.022) |
| GDP |  | -0.315 | -0.290 | -0.258 | -0.361 | -0.414 | 0.137 |
|  |  | (0.325) | (0.330) | (0.295) | (0.420) | (0.650) | (1.269) |
| % pop. with tertiary education |  |  | 0.004 | 0.007 | 0.006 | 0.005 | 0.010 |
|  |  |  | (0.014) | (0.013) | (0.012) | (0.011) | (0.012) |
| Broadband availability in business properties |  |  |  | -0.395*** | -0.404*** | -0.404*** | -0.067 |
|  |  |  |  | (0.112) | (0.118) | (0.117) | (0.441) |
| Unemployment benefit recipients |  |  |  |  | -0.017 | -0.025 | 0.091 |
|  |  |  |  |  | (0.041) | (0.066) | (0.196) |
| % large industrial firms |  |  |  |  |  | -0.284 | 1.921 |
|  |  |  |  |  |  | (1.442) | (4.216) |
| Log(population) in region |  |  |  |  |  |  | 3.072 |
|  |  |  |  |  |  |  | (4.148) |
|  |  |  |  |  |  |  |  |
| N | 10,051 | 10,051 | 10,051 | 10,051 | 10,051 | 10,051 | 10,051 |
|  |  |  |  |  |  |  |  |

Robust standard errors in parentheses are clustered for workers in the same region and industry. The table present results from OLS estimations. * p<0.10, ** p<0.05, *** p<0.01

**Table A14: Controlling for robot adoption in neighboring regions and industries. First stage results. Dependent variable: machine replacement.**

|  | (1) | (2) | (3) | (4) |
| --- | --- | --- | --- | --- |
|  |  |  |  |  |
|  |  |  |  |  |
| Robot adoption | 0.831*** | 2.188*** | 0.832*** | 2.190*** |
|  | (0.270) | (0.720) | (0.270) | (0.719) |
| Robot adoption in other industries | 2.024 | 5.698 |  |  |
|  | (2.609) | (6.990) |  |  |
| Robot adoption in other regions |  |  | -3.251 | -8.937 |
|  |  |  | (3.453) | (9.290) |
| Age | -0.004*** | -0.011*** | -0.004*** | -0.011*** |
|  | (0.001) | (0.002) | (0.001) | (0.002) |
| Union membership | -0.056*** | -0.149*** | -0.056*** | -0.149*** |
|  | (0.013) | (0.036) | (0.013) | (0.036) |
| Income scale = 2 | -0.094*** | -0.269*** | -0.094*** | -0.269*** |
|  | (0.024) | (0.065) | (0.024) | (0.065) |
| Income scale = 3 | -0.006 | -0.019 | -0.006 | -0.018 |
|  | (0.022) | (0.057) | (0.022) | (0.057) |
| Income scale = 4 | 0.024 | 0.062 | 0.024 | 0.062 |
|  | (0.023) | (0.060) | (0.023) | (0.060) |
| Income scale = 5 | 0.027 | 0.072 | 0.027 | 0.072 |
|  | (0.029) | (0.077) | (0.029) | (0.077) |
| Income scale = 6 | 0.043 | 0.112 | 0.043 | 0.112 |
|  | (0.034) | (0.090) | (0.034) | (0.090) |
| Income scale = 7 | 0.078*** | 0.205*** | 0.078*** | 0.205*** |
|  | (0.026) | (0.068) | (0.026) | (0.068) |
| Income scale = 8 | 0.039 | 0.100 | 0.039 | 0.100 |
|  | (0.026) | (0.070) | (0.026) | (0.070) |
| Income scale = 9 | 0.069 | 0.182 | 0.069 | 0.182 |
|  | (0.053) | (0.137) | (0.053) | (0.137) |
| University degree | 0.037* | 0.098* | 0.037* | 0.098* |
|  | (0.021) | (0.055) | (0.021) | (0.055) |
| Woman | -0.011 | -0.030 | -0.011 | -0.030 |
|  | (0.017) | (0.045) | (0.017) | (0.045) |
| Industry employment | -0.044** | -0.117*** | -0.044** | -0.118*** |
|  | (0.016) | (0.043) | (0.016) | (0.043) |
|  |  |  |  |  |
| Controls |  |  |  |  |
| Regional dummies | ✓ | ✓ | ✓ | ✓ |
| Year dummies | ✓ | ✓ | ✓ | ✓ |
|  |  |  |  |  |
| F-stat | 9.47 |  | 9.48 |  |
|  |  |  |  |  |
| N | 10,051 | 10,051 | 10,051 | 10,051 |

Robust standard errors in parentheses are clustered for workers in the same region and industry. Columns 1 and 2 present OLS estimates. Columns 3 and 4 show probit estimates. * p<0.10, ** p<0.05, ***p<0.01

**Table A15: First and second stage results. Adding interaction variables between union membership, robot adoption and machine replacement.**

|  | (1) | (2) | (3) | (4) |
| --- | --- | --- | --- | --- |
|  |  |  |  |  |
|  | OLS | Probit | 2SLS | IVOPROBIT |
|  | 1^st^ stage | 1^st^ stage | 2^nd^ stage | 2^nd^ stage |
|  |  |  |  |  |
| Robot adoption | 0.841* | 2.160* |  |  |
|  | (0.456) | (1.183) |  |  |
| Robot adoption X Union | 0.019 | 0.141 |  |  |
|  | (0.457) | (1.168) |  |  |
| Machine replacement |  |  | -0.674 | -0.393*** |
|  |  |  | (0.520) | (0.110) |
| Machine replacement X Union |  |  | -0.128 | -0.576 |
|  |  |  | (0.502) | (0.378) |
| Age | -0.004*** | -0.011*** | 0.007*** | 0.009*** |
|  | (0.001) | (0.002) | (0.002) | (0.002) |
| Union membership | -0.057** | -0.157** | 0.013 | 0.197 |
|  | (0.026) | (0.070) | (0.220) | (0.148) |
| Income scale = 2 | -0.093*** | -0.268*** | -0.133** | -0.111* |
|  | (0.024) | (0.065) | (0.068) | (0.061) |
| Income scale = 3 | -0.006 | -0.019 | 0.010 | 0.023 |
|  | (0.021) | (0.056) | (0.037) | (0.041) |
| Income scale = 4 | 0.024 | 0.063 | 0.093* | 0.119** |
|  | (0.022) | (0.059) | (0.053) | (0.054) |
| Income scale = 5 | 0.027 | 0.072 | 0.143*** | 0.182*** |
|  | (0.029) | (0.077) | (0.043) | (0.045) |
| Income scale = 6 | 0.043 | 0.112 | 0.194*** | 0.237*** |
|  | (0.034) | (0.090) | (0.057) | (0.066) |
| Income scale = 7 | 0.078*** | 0.205*** | 0.213*** | 0.272*** |
|  | (0.026) | (0.070) | (0.049) | (0.055) |
| Income scale = 8 | 0.039 | 0.100 | 0.200*** | 0.257*** |
|  | (0.026) | (0.070) | (0.060) | (0.076) |
| Income scale = 9 | 0.069 | 0.181 | 0.319*** | 0.414*** |
|  | (0.053) | (0.138) | (0.070) | (0.092) |
| University degree | 0.037* | 0.098* | 0.034 | 0.020 |
|  | (0.021) | (0.056) | (0.026) | (0.027) |
| Woman | -0.011 | -0.030 | 0.090*** | 0.126*** |
|  | (0.017) | (0.044) | (0.018) | (0.032) |
| Industry employment | -0.044** | -0.118*** | -0.169*** | -0.213*** |
|  | (0.016) | (0.044) | (0.020) | (0.025) |
|  |  |  |  |  |
| Controls |  |  |  |  |
| Regional dummies | ✓ | ✓ | ✓ | ✓ |
| Year dummies | ✓ | ✓ | ✓ | ✓ |
|  |  |  |  |  |
| N | 10,051 | 10,051 | 10,051 | 10,051 |

Robust standard errors in parentheses are clustered for workers in the same region and industry. * p<0.10, ** p<0.05, *** p<0.01
